# A test of ecophysiological theories on tropical forest functional traits along a VPD gradient

**DOI:** 10.1038/s42003-025-08420-1

**Published:** 2025-07-09

**Authors:** Huanyuan Zhang-Zheng, Yadvinder Malhi, Kasia Ziemińska, Agne Gvozdevaite, Theresa Peprah, Mickey Boakye, Stephen Adu-Bredu, Jesús Aguirre-Gutiérrez, Sam Moore, David Sandoval, Minxue Tang, Iain Colin Prentice, Imma Oliveras Menor

**Affiliations:** 1 Environmental Change Institute, School of Geography and the Environment, University of Oxford, Oxford, UK; 2 Leverhulme Centre for Nature Recovery, University of Oxford, Oxford, UK; 3 Forestry Research Institute of Ghana, Council for Scientific and Industrial Research, Kumasi, Ghana; 4 Department of Environmental Science, Policy, and Management, University of California, Berkeley, CA USA; 5 Department of Life Sciences, Georgina Mace Centre for the Living Planet, Imperial College London, Silwood Park Campus, Ascot, UK; 6 AMAP – botAnique et Modélisation de l’Architecture des Plantes et des Végétations, Université de Montpellier, CIRAD, CNRS, INRAE, IRD, Montpellier, France

**Keywords:** Forest ecology, Plant physiology

## Abstract

Forest primary production is a crucial process for both ecosystem functioning and global carbon cycling. Primary production responds to both temperature and vapour pressure deficit (VPD) through separate mechanisms. Vegetation models need to quantify both responses. However, due to their often high correlations, most observational data sets used to test models or theories hardly distinguish them. Here we evaluate ecophysiological theories on the effect of VPD using tree trait data collected along a VPD gradient in West Africa. Study sites spanned an annual rainfall range of 1200–2050 mm, with varying seasonality but minimal temperature variation. Most photosynthetic traits show trends consistent with predictions from optimality theory, including higher net CO2 assimilation rates and greater photosynthetic capacity at drier sites. These patterns were associated with greater deciduousness, increased respiration rates and enhanced water transport at drier sites. In contrast, hydraulic traits showed weaker consistency with theoretical predictions or global trends, particularly those based on the xylem efficiency-safety tradeoff. Our findings suggest that vegetation models should account for higher photosynthetic capacity in drier regions, but that further research is needed to incorporate hydraulic traits into models.

## Abstract (web summary)

Testing ecophysiological theory using tree traits along a vapour pressure deficit (VPD) gradient shows, as predicted, that photosynthetic capacity is higher at higher VPD sites. Contrary to the predictions, potential water conductivity tends to increase with higher VPD.

## Introduction

Understanding how environmental factors influence forest photosynthesis is crucial for predicting their responses to climate change. Three key photosynthetic processes are frequently considered when seeking to understand plant photosynthesis: light availability and electron transport; air dryness and water transport; and CO_2_ concentration and carboxylation^1^. Plants’ capacities in these photosynthetic processes vary considerably along environmental gradients^2–5^. Recently, many efforts have been made to propose universal rules to explain worldwide plant photosynthetic patterns. For example, “optimality theory” was developed with the assumption that plants can optimize photosynthesis and minimize maintenance costs according to their living environments, which was used recently to provide a universal prediction of plant photosynthesis under different growing environments^6–11^.

One of the main challenges confronting these universal rules is explaining the effect of atmospheric dryness on photosynthesis^12^, especially in the tropics, where vapor pressure deficit (VPD) varies considerably more than temperature. Such challenges become particularly pressing in the context of climate change, as greater VPD is predicted for most tropical places^13–15^, which may strongly influence photosynthesis and hence the carbon cycle^16^. Although in previous studies, many universal ecophysiological theories have been shown to successfully explain photosynthetic patterns on multiple spatial scales^17–19^, most studied systems have substantial growing-season temperature variation. Temperature is an important driver of photosynthetic traits^20,21^, which could overshadow the effect of VPD, and little attention has been paid to trait variation along VPD gradients^14^. It is thus not known whether these theories would hold in the tropics, where VPD variation is more prominent than temperature.

The primary evolutionary adjustments to a drier environment are reduced stand density (fewer trees) and deciduousness (shorter leaf duration). Additionally, photosynthesis per unit leaf area may vary spatially from wet to dry sites, where many theories have been proposed and tested on a global scale^18^. For example, “optimality theory” hypothesized that compared to wet forests, dry forests should have a higher electron-transport capacity standardized to 25 °C (*J*_max25_) and higher Rubisco carboxylation capacity standardized to 25 °C (*V*_cmax25_) (see more predictions in “Methods”). However, the effect of VPD and the reliability of these theories within tropical forests have been less tested using field measurements. Within the tropical forest biome, most current earth system models simulate a negative relationship between photosynthesis (measured by CO_2_ assimilation rate per leaf area, *A*_area_) and VPD simply due to the closing of stomata without incorporating the dynamics of photosynthetic capacity within the tropic (measured by *J*_max25_ and *V*_cmax25_)^4,22^, but on a global scale higher *V*_cmax25_ was indeed found for plants grown in drier sites^21,23,24^. To incorporate tropical forests’ photosynthesis patterns in models, two particular challenges need to be clarified. First, how functional traits associated with photosynthesis vary along a VPD gradient within the tropical forest biome. Second, optimality theory predicted higher *V*_cmax_ and *A*_area_ under higher VPD^20^, which implies higher transpiration, but it is unclear how plants in drier environments arrange water uptake to meet greater transpiration demand^10^. Specifically, a previous study^8^ links photosynthesis with a plant’s hydraulic system. The study rested on the idea that water loss through stomata must equal water transport through xylem, assuming no change in stored water in the xylem on an annual scale:

1 $$\frac{E}{{A}_{L}}=\frac{1.6\cdot {{\mbox{VPD}}}_{{la}}\cdot {A}_{{\mathrm{area}}}}{\left({c}_{a}-{c}_{i}\right)\cdot {P}_{{\mathrm{atm}}}}={K}_{s}\cdot {\Delta \Psi }_{\max }\cdot \frac{{A}_{S}}{{A}_{L}}\cdot \frac{1}{h}$$

Where *E*/*A*_*L*_ is water transpired per leaf area surface (mol m^−2^ s^−1^), VPD_la_ is leaf-to-air VPD, *P*_atm_ is atmospheric pressure (MPa), *K*_*s*_ is sapwood-specific hydraulic conductivity (mol m^−1^ s^−1^ MPa^−1^), *A*_*S*_/*A*_*L*_ is the ratio of sapwood to leaf area (m^2^ m^−2^), Δ*Ψ*_max_ is the maximum decrease in water potential from soil to leaves (MPa), and h is the transpiration stream path length (m), roughly equivalent to plant height, 1.6 VPDla *A*_area_/(*c*_*a*_−*c*_*i*_)/*P*_atm_ denotes “water loss through stomata,” and *K*_*s*_ Δ*Ψ*_max_ *A*_*S*_/*A*_*L*_/*h* denotes water transport through xylem. *A*_area_ is the CO_2_ assimilation rate per leaf area (μmol CO_2_ m^−2^ s^−1^), and leaf internal (*c*_*i*_, ppm) and external (*c*_*a*_, ppm) CO_2_ concentration.

As explained above, *E*/*A*_*L*_ is greater in drier environments. However, it is unknown how tropical forests adjust *K*_*s*_ ΔΨmax *A*_*S*_/*A*_L_/*h* in response to greater *E*/*A*_*L*_ toward drier sites.

To address these questions, we measured tree functional traits associated with photosynthesis and water transport along an environmental gradient in West Africa spanning woody savannas to wet-evergreen forests. Along this gradient, sites ranged considerably in VPD and light availability but less so in temperature. Based on ecophysiological theories proposed in previous literature, we developed 14 hypotheses (Table 1), which are explained in “Methods.” These hypotheses were tested using detailed trait measurements. Specifically, we addressed key ecological questions: (1) Do forests in drier environments have higher photosynthesis rates (indicated by *A*_sat400_ and *A*_sat2000_) and how do VPD and photosynthetic traits interact? (Hypotheses 1–6, Table 1) (2) How do forests meet greater transpiration demand per leaf area in drier sites? (Hypotheses 8–14, Table 1). If field measurements are consistent with the theoretical predictions, this would provide a mechanistic explanation for the impact of VPD on the specific trait. In contrast, if field measurements are inconsistent with the theoretical predictions, this would imply either a weak effect of VPD on the specific trait or that the theory needs to be revised. Overall, we found that all investigated photosynthetic traits show a clear spatial variation from wet to dry sites, consistent with theoretical hypotheses. In contrast, hydraulic traits surprisingly show weaker spatial variation from wet to dry sites. Data-theory inconsistencies were found in some hydraulic traits. Our study highlights the importance of incorporating higher photosynthetic capacity for drier tropical forests when modelling forest productivity, while also underscoring the need for further refinement of hydraulic trait theory.

**Table 1:**
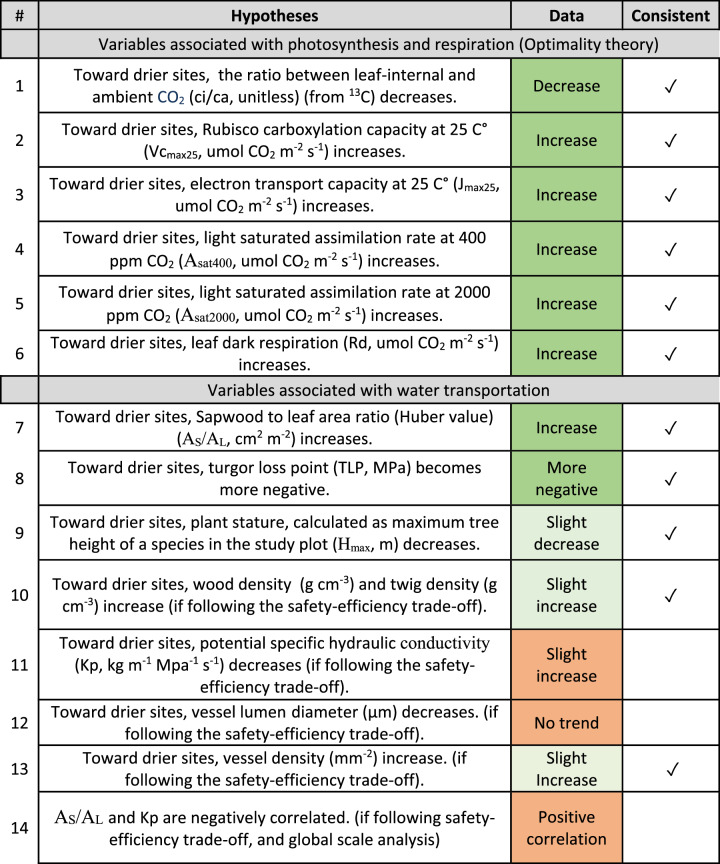
Traits name, unit, hypotheses, and findings from field measurements along the VPD gradient

“Data” column summarizes patterns in Figs. 3 and 4. A trend of trait is qualitatively recognized if KOG (dry region) is significantly different from ANK (wet region) while BOB ranks between. “Slight increase” suggests that the pattern fits the above criteria broadly, albeit one plot behaves inconsistently. Colors indicate results that are consistent (green), weakly consistent (light green), and inconsistent (orange) with theoretical expectations. Ticks in the column “consistent” indicate consistency between hypotheses and data.

## Results

### Leaf area index and deciduousness

**Fig. 1:**
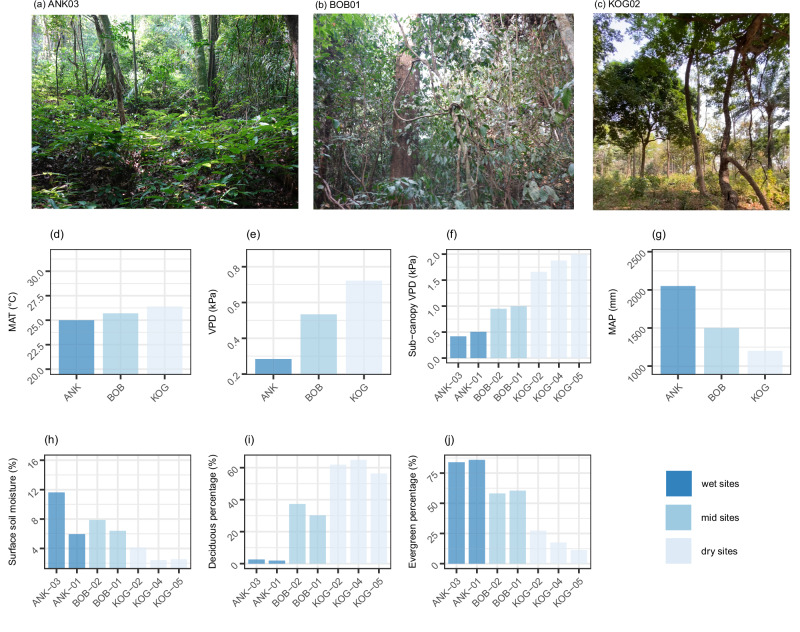
Study sites environment and characteristics. There are seven study one-hectare plots, grouped in three sites (ANK, BOB, and KOG), from wet (left, dark blue) to dry (right, light blue). We show field photos taken in January 2022 (dry season) for plot **a** ANK03, **b** BOB01, and **c** KOG02. We also show **d** mean annual air temperature (MAT, °C); **e** vapor pressure deficit (VPD, kPa), **f** daytime sub-canopy VPD (kPa), **g** mean annual precipitation (MAP, mm/year) and **h** soil volumetric water content at 12 cm depth (Soil moisture) as annual mean (%). VPD, MAT, and MAP are measured by meteorological stations. Sub-canopy VPD is daytime vapor pressure deficit beneath the canopy measured during June–December 2014 using a handheld hygrometer (see “Methods”). The figure also shows **i** the percentage of deciduous trees (%) and **j** the percentage of evergreen trees (%) at each plot. The sum of deciduous and evergreen is less than 100% because we are uncertain about the phenology of a handful of species. Image (**c**) has been reproduced from a previous publication^57^.

**Fig. 2:**
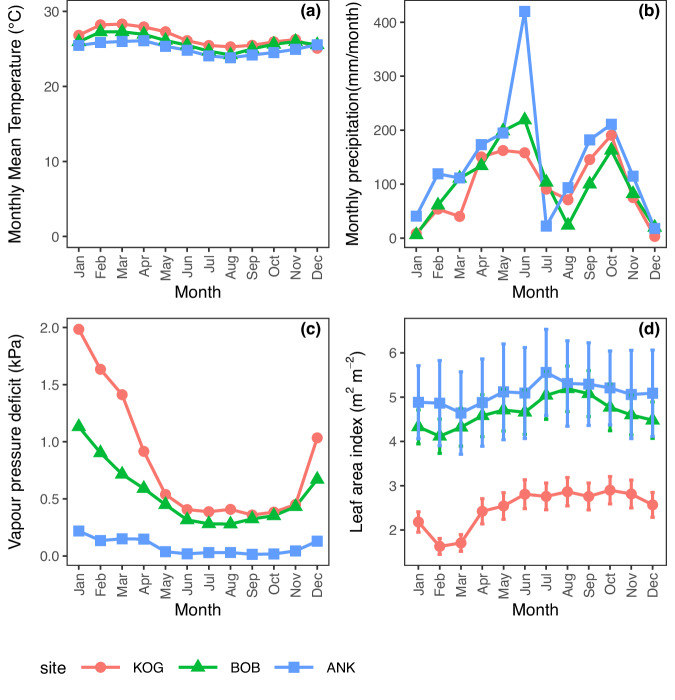
Study site seasonality. We show **a** monthly mean temperature (°C), **b** monthly sum precipitation (mm/month), **c** vapor pressure deficit (kPa), and **d** leaf area index (m^2^ m^−2^) for the three study sites. The error bar on Leaf area index denotes systematic error—uncertainty in measurements and calculation method.

The VPD gradient consists of seven plots, clustered in three regions (ANK, BOB, and KOG). From wet to dry sites, the mean annual temperature increases slightly (25–26.4 °C), while the mean annual VPD rises from 0.28 to 0.72 kPa. Annual rainfall ranges from 1200 mm to 2050 mm. The plot-to-plot variation of sub-canopy VPD and surface soil moisture is very similar, which was used to rank plots from wettest to driest (Fig. 1). Regarding seasonality, both climate seasonality and deciduousness increase toward high-VPD sites (Fig. 2). All study plots are semi-deciduous, meaning that they contain both evergreen and deciduous tree species. From wet to dry sites, the percentage of deciduous tree individuals (rather than species) increases from 2% to 65%. There is no seasonal variation in temperature (Fig. 2). All study sites experience two rainy seasons in similar months, though mean annual precipitation ranges from 2050 to 1200 mm. However, in terms of VPD seasonality, there is only one dry season (from December to February) across all study sites—unlike rainfall seasonality—because the no-rain periods in July and August remain humid and cloudy. The Leaf Area Index (LAI) at sites ANK and BOB shows slight seasonal fluctuations, ranging from 4.1 and 5.5. In contrast, KOG exhibits more pronounced seasonality, with LAI varying from 1.55 to 2.9. The study plot KOG05 loses most of its leaves only in February, while the other KOG plots retain some leaves throughout the year. Across all study sites, February and March are the months with relatively fewer leaves. At any time of the year, KOG has fewer leaves (almost half) than ANK and BOB.

### Traits variation along the VPD gradient

**Fig. 3:**
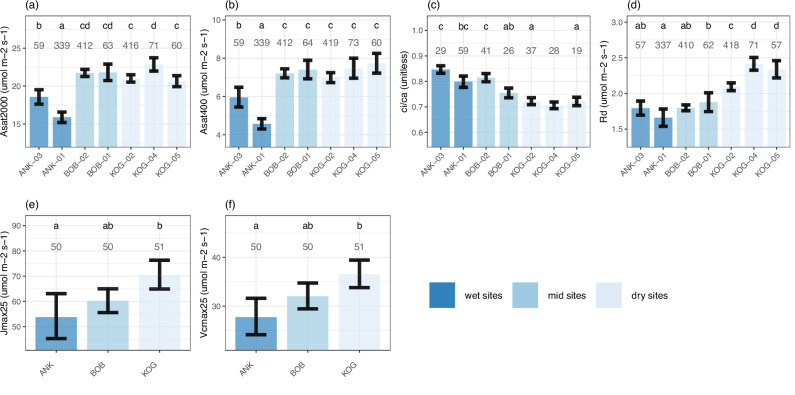
Plot scale community-weighted mean of measured traits (with standard error) associated with photosynthesis and respiration. The figure shows study plots from the wettest (left, dark blue) to the driest (right, light blue) plot. Forest plots are arrayed from left to right in order of VPD. The number denotes the number of samples, which could be a leaf, a branch, a tree, or a species, depending on the variable. The letters denote significance (ANOVA, *P* < 0.05) in plot-to-plot difference. The figure shows **a** light-saturated assimilation rate at 2000 ppm *A*_sat2000_ (μmol CO_2_ m^−2^ s^−1^), **b** light-saturated assimilation rate at 400 ppm *A*_sat400_ (μmol CO_2_ m^−2^ s^−1^), **c** the ratio between leaf-internal and ambient CO_2_ (*c*_*i*_/*c*_*a*_, unitless), **d** leaf dark respiration (*R*_*d*_, μmol CO_2_ m^−2^ s^−1^), **e** electron-transport capacity at 25 C° (*J*_max25_, μmol CO_2_ m^−2^ s^−1^), and **f** Rubisco carboxylation capacity at 25 C° (*V*_c__max25_, μmol CO_2_ m^−2^ s^−1^). Although data distributions are not shown in these panels, they are presented in Fig. S18.

Along the VPD gradient, we found consistency between theoretical prediction and field measurements (Table 1; Fig. 3) for all photosynthetic traits. Specifically, toward the dry forests site, *c*_*i*_/*c*_*a*_ decreases (from 0.85 to 0.71; hereafter, the numbers represent the lowest and highest plot-scale community-weighted mean), *V*_cmax25_ increases (from 22 to 46 µmol CO_2_ m^−2^ s^−1^), *J*_max25_ increases (from 38 to 91 µmol CO_2_ m^−2^ s^−1^), *R*_*d*_ increases (from 1.7 to 2.4 µmol CO_2_ m^−2^ s^−1^), *A*_sat400_ increases (from 4.6 to 7.7 µmol CO_2_ m^−2^ s^−1^), and *A*_sat2000_ increases (from 15.9 to 22.9 µmol CO_2_ m^−2^ s^−1^). The trends of all photosynthetic traits are successfully predicted by optimality theories (see “Methods”), which are a group of principles assuming that plants can optimize photosynthesis while minimizing water loss and maintenance costs according to their living environments^17^. Meanwhile, leaf economic spectrum traits (Fig. S2) and soil nutrients (Table S1) do vary among the study sites, which may also affect photosynthetic traits, but the effect of nutrient cycling on photosynthetic traits is out of the scope of this study.

**Fig. 4:**
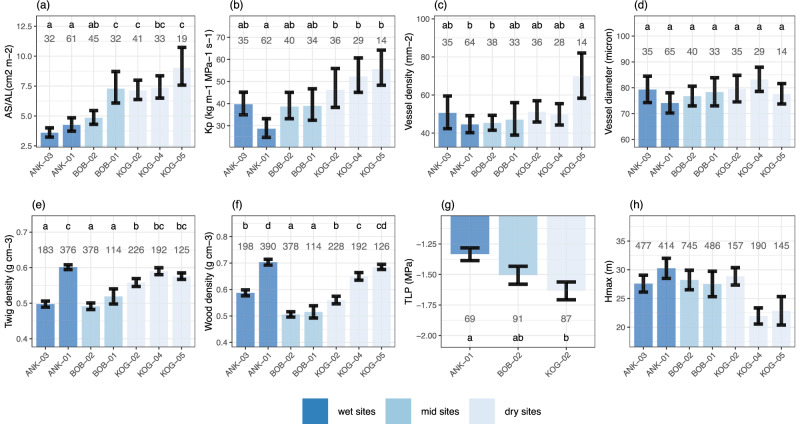
Plot scale community-weighted mean of measured traits (with standard error) associated with the plant’s hydraulic system. The figure shows study plots from the wettest (left, dark blue) to the driest (right, light blue) plot. Forest plots are arrayed from left to right in order of VPD. The number denotes the number of samples, which could be a leaf, a branch, a tree, or a species, depending on the variable. The letters denote significance (ANOVA, *P* < 0.05) in plot-to-plot difference. The figure shows **a** Sapwood to leaf area ratio (Huber value) (*A*_*S*_/*A*_*L*_, cm^2^ m^−2^), **b** potential specific hydraulic conductivity (*K*_*p*_, *k*_*g*_ m^−1^ Mpa^−1^ s^−1^), **c** vessel density (mm^−2^), **d** vessel lumen diameter (μm), **e** twig density (g cm^−3^), **f** wood density (g cm^−3^), **g** turgor loss point (TLP, MPa), and **h** plant stature, calculated as maximum tree height of a species in the study plot (*H*_max_, m). Statistics are not applied for *H*_max_ because every tree (>10 cm DBH) in the plot is measured. Although data distributions are not shown in these panels, they are presented in Fig. S19.

From a water transpiration perspective (Fig. 4), the hypotheses are consistent with field measurements for leaf traits. *A*_*S*_/*A*_*L*_ is higher in high-VPD sites (360–902 cm^2^ m^−2^), while TLP is more negative in high-VPD sites (−1.3 to −1.6 MPa). However, less consistency is found between theoretical expectations and field measurements for xylem-related traits. Along the VPD gradient, there is an increasing trend of field *K*_*P*_ toward drier sites (from 29 to 59 kg m^−1^ Mpa^−1^ s^−1^), which contradicts the xylem safety-efficiency trade-off. The trend is driven by vessel lumen diameter, which also contradicts the hypotheses. Vessel lumen diameter does not change along the VPD gradient, while vessel density increases toward drier sites (from 45 to 70 mm^−2^). The theory expects lower *K*_*P*_ and hence higher wood density toward drier sites, but the drier plots (KOG04, KOG05) have higher *K*_*P*_, higher twig density, and higher wood density than the wettest site (ANK03). Meanwhile, we also find *K*_*P*_ negatively correlates with twig density on species scales (a Simpson’s paradox) (Fig. S7) (Supplementary Note 1). ANK01 has very high wood density and twig density, which breaks the increasing trend formed by other plots. *H*_max_ decreases from wet to dry forest sites as expected.

Using variance partitioning, we find that the plot-to-plot trends of all traits are dominated by interspecific rather than intraspecific variation (i.e., components [a] are smaller than [b] in Fig. S4) (indicating the change of species composition). The analogous patterns between twig and wood density along the VPD gradient also support a shift in species composition, since twig density was field-measured and wood density was retrieved from a global database by species^25^. Nonetheless, variance induced by intraspecific variation or measurement errors (component [d]) was large for many traits. Especially for turgor loss point, this component accounts for 95% of the variance, followed by *V*_cmax25_ (74% of the variance) and *J*_max25_ (66% of the variance). In short, intraspecific and interspecific variation are both major components, depending on the trait. The plot-to-plot pattern (Figs. 3 and 4) results from changing species composition because the common dominant species among plots are very few. Using general additive models (Table S2), we found that site-level differences explain the variance of most traits. After accounting for site-level variation, plot-to-plot differences have limited explanatory power (except *A*_sat2000_ and *R*_*d*_). For *A*_sat2000_, GAM shows that the site-to-site difference (*F* = 37.5) is less than the nested term (site, plot) (*F* = 105.1). *A*sat2000 at ANK01 is much smaller than that at ANK03 (Fig. 3), likely because ANK01 is a hilltop that receives more sunlight than ANK03 in a seasonally inundated valley. The nested term (site, plot) is also significant (*F* = 615) for Dark Respiration, although much less than the site-to-site difference (*F* = 3083). Given that the nested terms are significant for these two traits, we also show these two traits on site scales (Fig. S5). Results from general additive models (Table S2) are consistent with those from ANOVA (Figs. 3 and 4), with both showing that all photosynthesis-related traits exhibit significant spatial patterns, but not traits associated with water transport.

### The coordination between photosynthesis and water transportation

**Fig. 5:**
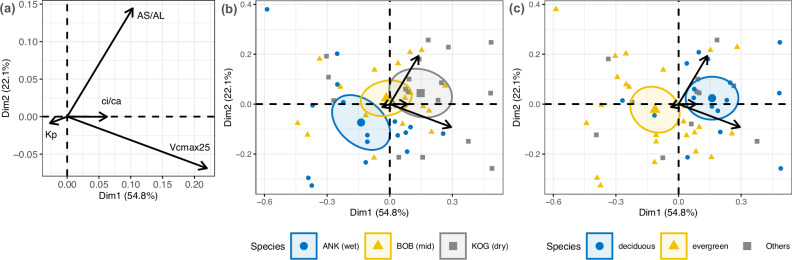
Principal components analysis for Huber value (*A*_*S*_/*A*_*L*_), the ratio between leaf internal and ambient CO_2_ (*c*_*i*_/*c*_*a*_), Rubisco carboxylation capacity at 25° (*V*_cmax25_), and potential specific conductivity (*K*_*p*_) on species scale (each point represents a species, containing measurements from multiple individuals). Values are transformed to achieve normal distribution but not standardized to equal variance; therefore, the length of arrows denotes the variance of the specific trait. The ellipses for each site are confidence ellipses around group mean points. The three panels display the same PCA, but with a different classification of scatter points. **a** The PCA without scatter points, but with labels for traits. **b** Scatter points according to study sites. **c** Scatter points according to phenology.

Data from our West African VPD gradient reveal a weak positive correlation between *K*_*p*_ and *A*_*S*_/*A*_*L*_, which contradicts Hypothesis 14 and is inconsistent with the negative correlation observed on global scales (Fig. S3). *A*_*S*_/*A*_*L*_ for the Ghanaian VPD gradient is higher than the global tropical average. Following Eq. 1, we further explore the link between *A*_*S*_/*A*_*L*_, *K*_*p*_ and photosynthetic traits. Species with both high *A*_*S*_/*A*_*L*_ and *K*_*p*_ tend to have higher *V*_cmax25_ and lower *c*_*i*_/*c*_*a*_. Such species tend to be deciduous and occur more in the drier forest plots (Fig. 5). There is greater variance of hydraulic traits compared to photosynthetic traits. The pattern remains consistent if we repeat the above Principal Component Analysis (PCA) with *A*_sat400_ instead of *V*_cmax25_ (Fig. S3). This finding supports Eq. 1.

## Discussion

Although most hypotheses (Table 1) have been tested with spatially varying VPD at global scales^17^, testing them along a tropical VPD gradient reveals the pattern without temperature variation. The following discussion considers community-weighted mean values of traits, disregarding individual-level patterns and seasonal variation. The patterns of all photosynthetic traits measured along the VPD gradient (*c*_*i*_/*c*_*a*_, *J*_max25_, *V*_cmax25_, *R*_*d*_, *A*_sat400_, *A*_sat2000_; namely, Hypotheses 1–7) are consistent with theoretical expectations, underscoring that VPD is a direct and critical driver of photosynthetic traits without confounding effects from temperature. The increase in photosynthetic capacity towards drier tropical forest sites helps multiple previous observations: (1) Savanna has higher *A*_sat400_ than wet-evergreen forest^5,26^; (2) In the tropics, drier sites are brighter and warmer, where higher photosynthetic capacity implies higher actual CO_2_ assimilation per leaf area. This explains why woody savannas have sparse canopies but similar gross primary productivity to wet-evergreen forest^27^. Toward the dry end of the gradient, although mean annual leaf area index decreases (from about 5.0 to 2.5 m^2^ m^−2^; Fig. 2), the photosynthesis rate per leaf area increases (Fig. 3). (3) For wet Amazonian forests, leaves flushed in dry season have higher photosynthetic capacities, which increase forest productivity ^22,28^; and (4) Tropical deciduous species have more acquisitive traits than tropical evergreen species^29,30^.

From a water transportation perspective, forests in drier sites are more deciduous (i.e., shorter leaf duration), which avoids the driest month (February) altogether (Fig. 2). Besides, forests at the periodically dry site have more negative TLP, lower *H*_max_, and higher *A*_*S*_/*A*_*L*_, supporting a greater mid-day transpiration stream (consistent with Hypotheses 8–10). However, we found slightly higher *K*_*P*_ toward drier sites, which is inconsistent with hypotheses derived from the safety-efficiency trade-off. First, this could be associated with the difference between *K*_*P*_ and *K*_*s*_—since vessels that become embolized in drier sites are not detected by anatomical images, and not all sapwood conducts water^31^. The much higher deciduousness in KOG (dry forests) than in the wet sites may play a role, as higher hydraulic efficiency has been observed from deciduous species or more deciduous forests^32–34^, since they need less hydraulic safety^35^. Furthermore, for environments with wet soils and dry atmospheres, high hydraulic efficiency has been observed, which reduces xylem water potentials and thus avoids harmful tension altogether^36^. The other reason may lie in geographic sampling bias. Previous studies used global datasets with scarce data points from West Africa, while we analyzed a Ghanaian dataset. We reported a negative correlation between *A*_*S*_/*A*_*L*_ and *K*_*p*_ at global scales but a positive correlation along the VPD gradient (Figs. S6 and S8). On a global scale, there is a confounding effect of temperature or vegetation type; for example, a negative correlation between *A*_*S*_/*A*_*L*_ and *K*_*s*_ was reported globally^37^ and on continental (Australia) scales^38^, but an insignificant correlation was also reported for tropical forest stands on local scales without varying temperature^39–41^. The above highlights that: (1) A global trait-trait relationship may not hold at local scales; (2) There is variation in hydraulic architecture^42^ across vascular plants, indicating that plants in relatively dry tropical forests have evolved strategies for many aspects of the hydraulic trade-off, such as deciduousness, deep roots that access belowground water^43^, and the cavitation repair capacity^44^. However, we did not measure such data along the VPD gradient, which limits further interpretation. Nonetheless, as xylem-related predictions show theory-data inconsistencies (Table 1), further investigations into xylem functioning are required to better understand how greater water transportation is achieved in drier sites.

Traits with the strongest VPD-gradient trends are *c*_*i*_/*c*_*a*_ (stomatal behavior), TLP (drought tolerance), and *A*_*S*_/*A*_*L*_ (water delivery), *presumably having closest* association with VPD (Table 1). Secondary influential traits include R_d_, R_stem_leaf_, *J*_max25,_ and *V*_cmax25_, known to acclimate to both *c*_*i*_/*c*_*a*_ and light intensity^9^. Although *c*_*i*_/*c*_*a*_, *V*_cmax25_, *K*_*P*_, and *A*_*S*_/*A*_*L*_ all vary along the VPD gradient, we further illustrate that, surprisingly, it is photosynthetic traits instead of hydraulic traits that contrast species from wet to dry forest sites (and from evergreen to deciduous) (Fig. 5). Given that the large photosynthetic traits variation from wet to dry forest plots was induced by a shift in tree species composition (Fig. S4), our study on a spatial scale agrees with a previous study on a temporal scale, which shows that in response to a drier climate, these forests change species composition with more deciduousness and higher *A*_sat400_^45^. Nonetheless, the above study did not incorporate intraspecific variation. Key photosynthetic traits (*A*_sat400_, *A*_sat2000_, and *V*_cmax_) have high intraspecific variation (Fig. S4), and the response of which to a changing climate would require future investigation. The study highlights that photosynthetic traits have substantial spatial variation from wet to dry sites within the tropical forest biome. The positive effect of VPD on *A*_sat400_, *A*_sat2000_, and *V*_cmax_ should be considered in vegetation models if these parameters are used to calculate forest productivity.

Overall, optimality theory can effectively explain plant photosynthetic trait variation along the VPD gradient. As theoretically expected (Eq. 1), species in drier sites (with greater deciduousness) tend to have reduced stomatal openness (*c*_*i*_/*c*_*a*_), greater photosynthetic capacities (*J*_max25_ and *V*_cmax25_) with higher maintenance cost (higher *R*_*d*_), higher photosynthesis rates (*A*_sat400_), and greater maximum transpiration per leaf area, supported by higher *K*_*P*_ and greater *A*_*S*_/*A*_*L*_. The product of *A*_*S*_/*A*_*L*_ and *K*_*P*_ is a proxy for water delivery per leaf area, which was previously found strongly correlated with proxies of photosynthesis rate: *A*_sat400_^46^, the quantum yield of electron transport^47^, and electron transfer rate^48^—consistent with this study (Fig. 5).

It appears that deciduousness is a key factor behind the spatial pattern reported in Table 1. Deciduousness increases along the VPD gradient in our study ecosystems (Fig. 5). A previous study reported a short leaf lifespan (ca. 6 months) in KOG (the periodically dry forest site)^49^, which is consistent with its high *A*_sat400_ (Fig. 3)^50^. Such “drought-avoiding” species can optimize their traits exclusively to the wet season when they focus on hydraulic efficiency (not safety) to support high photosynthetic activity^51^. It was shown that phenological differences lead to species from dry tropical forests exhibiting higher hydraulic conductivity and photosynthetic capacity than species from wet tropical forests^46,52,53^. The large variance of wood traits (considerably larger than that of leaf photosynthetic traits)^37^ (Fig. 5) suggests that plants might have a wide range of trait combinations to provide adequate water transport^7,54^ in drier sites to support faster photosynthesis. At least at our periodically dry sites (KOG), plants are not limited by water transport. At this site, the forest produces fewer leaves per unit land area (small LAI) and per sapwood area (*A*_*S*_/*A*_*L*_), but retains highly active leaves, rather than investing in abundant foliage and shutting it down for extended periods due to water shortage. The study thus highlights the central role of LAI seasonality (i.e., *A*_*S*_/*A*_*L*_ and deciduousness) in controlling water relations and facilitating high Asat at relatively dry sites.

In summary, this study derives 14 hypotheses from widely used ecophysiological theories pertaining to photosynthesis and anatomical proxies of water transport and tests them in a tropical setting. We report both consistencies and inconsistencies between theory and data. To summarize plant photosynthetic patterns along the tropical VPD gradient: a drier environment (while other environmental variables remain unchanged) leads to higher *V*_cmax25_ and lower *c*_*i*_/*c*_*a*_. Drier tropical forests typically receive more sunlight, resulting in higher *J*_max25_ and thus higher *A*_sat400_ and *A*_sat2000_. In terms of plant hydraulics, we found that species with higher photosynthetic rates tend to have higher *A*_*S*_/*A*_*L*_ and *K*_*P*_ (greater mid-day transpiration per unit leaf area) and are more deciduous (less leaf area in the dry season). With this working example in West Africa, the study underscores the importance of accounting for higher photosynthetic capacity at drier sites in carbon modeling, which could be embodied by linking *V*_cmax25_ to dryness indicators such as VPD, *c*_*i*_/*c*_*a*_, or rainfall. The incorporation of these factors should include adequate simulation of seasonality or deciduousness. The study is limited in that several traits were repeatedly sampled across seasons, but some traits were measured only once in the dry season. The study also implies that considering plant hydraulics and soil or leaf nutrients would not significantly improve the simulation of tropical forest photosynthesis along the VPD gradient. However, since soil moisture and VPD co-vary along the VPD gradient and both can cause stomatal closure^55^ (Fig. 1), their effects are confounded in this study, requiring further investigation. Given the observed inconsistencies with xylem-related theories, future research should test plant hydraulic theories more rigorously in tropical settings.

## Methods

### Study sites

Trait data were collected from seven one-hectare plots distributed along a VPD gradient across three sites, Ankasa (ANK, two wet rainforest plots), Bobiri (BOB, two moist and semi-deciduous forest plots), and Kogyae (KOG, two dry forest plots and one woody savanna plot) in Ghana, West Africa (Figs. 1, S1 and S9–S17), as part of the Global Ecosystem Monitoring (GEM) network^56^. These sites form a typical tropical VPD gradient that shares very similar mean annual temperature but spans a steep gradient of VPD and sunlight (Fig. 1, Table S1). VPD, MAP, and maximum cumulative water deficit all reveal a clear wet-dry gradient from ANK (wet) to BOB (mid) and KOG (periodically dry). Light increases toward higher VPD sites due to less cloudiness (Table S1)^57^. Within any site, there are many common species between plots, but species composition (e.g., the top five abundant species) is very different. More information on the plots is available in Table S1 and in previous studies^58,59^. Field-measured gross and net primary productivity of these sites are available in previous studies^27,49^. To understand deciduousness, we classified species as “deciduous,” “evergreen,” and “others” (whose species or phenology could not be identified) (Fig. 1).

Climate data provided in Fig. 1 and Table S1 were recorded by meteorological stations at each site (one for each site). All climate data are reported as mean annual values. We have also reported sub-canopy VPD (microclimate, Fig. 1), which was measured beneath the canopy at breast height from June 2014 to December 2014 using a handheld hygrometer during daytime. Therefore, daytime sub-canopy VPD is much higher than the annual mean VPD (both day and night). The sub-canopy VPD provides a comparison between plots, but it shouldn’t be used for future meta-analysis. In this paper, we use and refer to the annual mean VPD reported by meteorological stations throughout. One-hectare plots (e.g., BOB02) within the same site (e.g., BOB) share very similar air temperature and precipitation as they are only several hundred meters apart. Sun leaves (the focus of this paper) would experience very similar VPD. However, sub-canopy microclimate, including both temperature and VPD, and surface soil moisture could vary between plots within a site, due to variations in soil properties, topography, and canopy density (Fig. 1). For presentation (Figs. 3 and 4), we arrange plots by VPD. Soil properties were field-measured in 2013 and 2014 (Table S1), as an average across 0–30 cm. Soil volumetric water content (vwc, %) was measured in the field every month in 2016, using a soil moisture sensor probe over the depth 0–12 cm.

### Functional trait data measurements

All trait data reported in this study were field-measured except for wood density, which was obtained from a global species database^25^. See Table 1 for a list of traits.

Leaf traits field campaigns were conducted using a standardized protocol^5^. Field campaigns were conducted in both dry and wet seasons for some traits, but for others, trait measurements were only taken once. For the seasonally dry site (KOG), there are still leaves in the driest month, so it is possible to sample in the dry season. Thus, the spatial comparison is fair without bias associated with the sampling month. In principle, we selected species that contributed to up to 80% of the basal area of each plot and sampled the three largest individuals for each species (exceptions are stated below). The actual number of samples can be found in Table S1. From each selected individual, we sampled a sunlit branch, and from each branch, we used three leaves and three wood segments to measure leaf and wood traits, respectively. Only sunlit leaves were used in this analysis because temperature and light conditions of the shade leaves vary considerably from plot to plot, which dilutes the focus on the effect of VPD. The leaf lifespan of our study plots is available in a previous paper^49^. To calculate the percentage of deciduous species, as an index for deciduousness, we identified each species as deciduous or evergreen by searching the literature and by consulting local botanist, but still a small number of species remain unidentified.

The ratio of leaf internal to ambient CO_2_ (*c*_*i*_/*c*_*a*_, unitless) was estimated from leaf δ^13^C measurements (the stable isotope ratio relative to a standard material). First, we calculated ∆^13^C—the difference between the leaf stable isotope ratio and the atmospheric stable isotope ratio—from δ^13^C at the given location and time^60^. We then derived *c*_*i*_/*c*_*a*_ from ∆^13^C using Equation 11 in Peng et al.^19^. Samples were taken once between October 2014 and February 2015.

For light-saturated assimilation rate at 400 ppm CO_2_ (*A*_sat400_, µmol CO_2_ m^−2^ s^−1^) and at 2000 ppm CO_2_ (*A*_sat2000_, µmol CO_2_ m^−2^ s^−1^), we immediately placed the cut branch in water and recut it. Three leaves per tree were selected for measuring *A*_sat400_ and *A*_sat2000_. These traits were measured using LI-6400XT (Li-Cor Inc., Lincoln, NE, USA). The photosynthetic photon flux density was set to 2000 µmol m^−2^ s^−1^. After measuring *A*_sat400_ and *A*_sat2000_, leaf dark respiration (*R*_*d*_) (µmol m^−2^ s^−1^) was measured for the same leaf by turning off the light (0 µmol m^−2^ s^−1^) and allowing for acclimatization. The block temperature was maintained at 30 °C throughout the sampling period, which was similar to the ambient air temperature. Additional details can be found in the supporting information of these papers^5,45^. These traits were sampled repeatedly, covering all seasons from October 2014 to September 2016.

To determine leaf mass per area (LMA, m^−2^ kg^−1^), nitrogen content by area (N_area_, g m^−2^), nitrogen content by mass (N_mass_, g/kg), phosphorus content by area (P_area_, g m^−2^), and phosphorus content by mass (P_mass_, g/kg), leaves were dried in an oven at 70 °C until reaching constant mass. Total leaf lamina area (cm^2^) was calculated by scanning images using NIH ImageJ (http://rsbweb.nih.gov/ij/) and a custom MATLAB script (https://github.com/bblonder/leafarea). LMA was calculated by dividing the dried leaf mass by its area. Part of these data were reported in previous studies^5,26^; however, the dataset in this study is not identical due to additional sampling. Samples were collected during multiple field campaigns from October 2014 to September 2016.

To measure the maximum rate of electron transport at 25 °C (*J*_max25_, µmol CO_2_ m^−2^ s^−1^) and maximum rate of carboxylation at 25 °C (*V*_cmax25_, µmol CO_2_ m^−2^ s^−1^), we sampled one individual tree per species per plot to generate A-Ci curves (photosynthetic response to changes in substomatal CO_2_ concentration, Ci). The CO_2_ concentration was changed in the following sequence: 400, 300, 200, 100, 500, 400, 600, 800, 1200, 1500, and 2000 µmol m^−2^ s^−1^, allowing >5 min for acclimatization at each level. The photosynthetic photon flux density was set at 2000 µmol m^−2^ s^−1^, and the block temperature was held constant near ambient temperature (30 °C). We employed the A-Ci curve fitting method, following the detailed procedure outlined in Appendix B of Domingues et al.^58^ to derive *V*_cmax_ and *J*_max_ values. To facilitate comparison with broader literature on photosynthetic capacity variability, we normalized the measured and estimated values of *V*_cmax_ and *J*_max_ to a reference temperature of 25 °C, following Sharkey et al.^61^. These temperature-scaled values are hereafter referred to as *V*_cmax25_ and *J*_max25_. Data collection for *V*_cmax25_ and *J*_max25_ was conducted once between October 2014 and February 2015. Due to incomplete fieldwork at plots ANK03, KOG04, and KOG05, sample sizes at these plots were limited. However, sampling was successfully completed at plots ANK01, BOB01, BOB02, and KOG02; we thus reported *V*_cmax25_ and *J*_max25_ at the site scale.

To calculate sapwood area-to-leaf area, or Huber value (*A*_*S*_/*A*_*L*_, cm^−2^ m^−2^), we first measured the leaf area (*A*_*L*_) of terminal, sun-exposed shoots from the outer canopy. Leaves were scanned using a Canon Lided220® flatbed scanner, and leaf area was quantified using a custom Matlab script (available at https://github.com/bblonder/leafarea). *A*_*S*_/*A*_*L*_ was calculated under the assumption that the branch diameter (bark excluded) corresponds to the sapwood area. Petioles were excluded from leaf area measurements. Sampling was conducted once between October 2014 and February 2015.

Wood anatomical traits were analyzed in cross-sections of one sun-exposed twig per individual (three replicates per species). Equation (1) can be interpreted at either the whole-plant or shoot level. However, whole-plant measurements are logistically challenging, so this focused on the shoot level. We used potential sapwood-specific hydraulic conductivity (*K*_*P*_) as a proxy for sapwood-specific hydraulic conductivity (*K*_*S*_). *K*_*P*_ was derived from vessel density and vessel lumen diameter^39^. Nonetheless, *K*_*P*_ and *K*_*S*_ may decouple because not all sapwood conducts water^31^. Additionally, twig *A*_*S*_/*A*_*L*_ served as a proxy for whole-tree *A*_*S*_/*A*_*L*_, and maximum plant height. We used plant stature (*H*_max_) as a proxy of path length (*h*). *H*_max_ is defined as the maximum tree height of each species, which was measured using a clinometer at the study sites. While *H*_max_ does not account for root depth and multi-layered canopy structure, this approximation was sufficient for hypothesis testing in the study region. However, caution is advised when applying this proxy in future modeling studies.

For the measurement of twig density (g cm^−3^), vessel density (n mm^−2^), and average vessel lumen diameter (μm), we sampled twigs (~8–10 mm diameter) with three replicates per species^26^. Cross-sections (20–50 μm thick) were prepared using a sliding microtome, stained with safranin O and alcian blue, and permanently mounted on microscope slides. A pie-shaped segment (pith to cambium) was photographed using an Optronics Microfire camera mounted on an Olympus BX-50 microscope with PictureFrame software. Vessels within the region were highlighted using the Magic Wand Tool (GIMP, http://gimp.org) and a Wacom Cintiq 22HD interactive pen display. Vessel area, average diameter (mean of minimum and maximum diameters), and pie-section area were measured in ImageJ. The average vessel lumen diameter (VD) was derived from the mean diameters of all vessels per given pie section and used in subsequent analyses. Vessel density (ρV) was calculated as the total vessel count divided by the pie-section area. Twigs were oven-dried (105 °C, ≥72 h), and dry mass was measured on a precision balance. Twig density was determined as dry mass divided by the volume of soaked wood. Sampling occurred once between October 2014 and February 2015.

For the turgor loss point, TLP (MPa), a sunlit branch with fully grown leaves was collected and recut while submerged in water. The branch was then rehydrated overnight, covered with a black plastic bag sprinkled with water on the inside, and left to rehydrate for ~15 h. Three mature leaves were collected from each branch to generate pressure-volume curves, following the method described by Maréchaux et al.^62^. PV curves were fitted to extract TLP using a custom script^63^. The pressure-volume curves were measured in several field campaigns from October 2014 to September 2016.

Wood density was provided by Forestplot.net, which compiled measurements from Zanne et al.^25^. For comparative analysis (Fig. S3), we also compared our data with *A*_*S*_/*A*_*L*_ from this study^37^ and vessel lumen diameter from Xylem Functional Traits Database^64^.

Leaf area index (LAI) was estimated from hemispherical images taken using a Nikon D5100 camera with a Nikon Fisheye Converter FC-E8 (0.21×) lens. Images were captured monthly during 2016–2017 (ANK) and 2012–2017 (BOG and KOG) at the center of each subplot (25 per plot) at a standardized height of 1 m, exclusively under overcast conditions. Following established protocols (Zhang et al.^65^ and GEM manual^56^), images were processed using ilastik software^66^ for pixel classification and CANEYE^67^ for LAI calculation. Key parameters in CANEYE included: COI = 80, Sub-sample factor = 1, Fcover = 20, and PAIsat = 10. Data were subsequently extracted and analyzed using R. For LAI calculation, we selected CANEYE’s most recent algorithm (CE V6.1 True PAI). Since systematic errors dominate LAI calculations, we quantified uncertainty as the standard deviation across all four LAI calculation methods (“CE V6.1,” “CE V5.1,” “Miller,” and “LAI2000”).

### Statistics and reproducibility

Hypotheses 1–13 (Table 1) were evaluated by testing for significant differences between wet and dry forest plots. For Hypothesis 14, we employed standardized major axis regression to examine relationships between *K*_*s*_, *A*_*S*_/*A*_*L*_, and photosynthesis traits. This method accounts for measurement uncertainty in both axes and was implemented using the smatr::sma() function^50,68^. This analysis evaluated both regression slopes and the statistical significance of correlations.

We performed a plot-to-plot comparison to address Hypotheses 1–13 as follows: (1) We visually inspected histograms of each trait and applied transformations to achieve normal distribution where necessary; (2) Outliers were checked with the R package *outliers::scores* using the interquartile range method (IQR) with a threshold of 1.5^69^. Extreme values were retained when confirmed to be error-free; (3) Community-weighted means were calculated based on species’ basal area values. Standard errors were calculated using the same weights^70^; (4) The significance of differences in plot-to-plot community-weighted means was tested with Tukey’s one-way ANOVA using the *multcomp* package^71^, with basal area as weights. For Hypotheses 1–13, we considered hypothesis accepted if KOG (periodically dry) differed significantly from ANK (wet) while BOB (mid) was intermediate (Figs. 3 and 4); and (5) Variance partitioning was performed with the *vegan* package using redundancy analysis ordination (RDA) with the expression: *varpart* (Trait, ~ Plot, ~ Species, data = Trait). This analysis reveals whether trait variation along the gradient was driven by intraspecific or interspecific variation. Note that plots within a site share common species (e.g., ANK01 to ANK03), but species composition differs substantially between sites (e.g., ANK01 to KOG02). Variance partitioning also helps determine whether the intraspecific variation or measurement errors dominate. To account for the nested structure of plots within sites, we used the *mgcv* package to fit generalized additive models, which compared plot-to-plot to site-to-site variation. The *mgcv* package also assesses whether the plot-to-plot variation is significant.

To illustrate the link between photosynthesis and hydraulics (Eq. 1), we used PCA with the R package *FactoMineR*^72^. To show the influence of deciduousness on the spatial pattern of traits, we colored points (tree species) in the PCA (Fig. 5) according to study sites and deciduousness. *A*_sat400_, *K*_*P*_, *A*_*S*_/*A*_*L*_, and *V*_cmax25_ were log10 transformed. To illustrate the degree of variance of each trait, we avoided scale standardization by disabling the “scale.unit” in the function PCA(), so that the variance of a trait was reflected by the length of its arrow in Fig. 5 (i.e., a longer arrow indicates greater variance in the dataset.

### Hypothetical deduction

In this section, we explain how we derive hypotheses in Table 1.

“Optimality theory” was developed with the assumption that plants can optimize photosynthesis and minimize maintenance costs according to their living environments, which was used recently to provide a universal prediction of plant’s photosynthesis patterns under different growing environments^6–11^. Although the above-cited studies have tested the theories on global scales and along elevation gradients, discussion and validation of these theories along the VPD gradient are still lacking. The following paragraph starts with photosynthetic traits and “Optimality theory” before consideration of plants’ hydraulics.

As explained in refs. ^7,73–75^, plants in dry sites maximize the carbon return per molecule of water by keeping stomata relatively closed. Thus, in dry sites, plants are expected to have a low leaf internal-to-external CO_2_ ratio (*c*_*i*_/*c*_*a*_) and low stomatal conductance (*g*_*s*_). The “coordination hypothesis”^76–78^ assumes equilibrium between Rubisco-limited photosynthesis rates (*A*_*C*_) (depending on *V*_cmax25_ and *c*_*i*_) and electron-transport-limited photosynthesis rates (*A*_*J*_) (depending on *J*_max25_ and leaf-absorbed photosynthetic photon flux density, PPFD) (see the quantitative expression in refs. ^6,9,20^). To maintain such an equilibrium, plants in dry sites are expected to have large *V*_cmax25_ to compensate for the low *c*_*i*_. Otherwise, *A*_*C*_ would be lower than *A*_*J*_ resulting in the waste of light (PPFD). To sum up, lower *c*_*i*_ but higher *V*_cmax25_ is expected toward drier sites if *J*_max25_ stays constant (in which case *A*_*J*_ would be slightly lower due to smaller *c*_*i*_).

In reality, toward drier sites, it is common to see higher leaf-absorbed photosynthetic photon flux density (*I*_abs_) because of less cloud cover and more open canopies. Considering an additional optimality criterion that *J*_max25_ is acclimated to *I*_abs_^20^, supported by multiple experiments^79,80^, we would expect high *J*_max25_ and *A*_*J*_ in dry sites, which further encourages high *V*_cmax25_ (see above paragraph). High *J*_max25_ would give rise to high *A*_*J*_, implying high *A*_*C*_ following the “coordination hypothesis.” All the above would lead to high leaf photosynthetic protein cost in dry sites, hence high leaf dark respiration (*R*_*d*_). Some of the above predictions have been seen on a global scale; for example, higher *R*_*d*_ has been found in drier sites than wetter sites^73,81^, and higher assimilation rate has been reported from drier sites^21,23,24,82^.

It is worth noting that *V*_cmax25_, *g*_*s*_, and *c*_*i*_ in this paper are discussed as an overall value for a forest stand, disregarding diurnal variation and intraspecific variation^83,84^. For instantaneous measurements, there is a positive correlation between *A*_sat400_ (light-saturated assimilation rate at 400 ppm), *V*_cmax25_, *g*_*s*_, and *c*_*i*_^7,74^, instead of the opposite trend of *V*_cmax25_ and *c*_*i*_/*c*_*a*_ discussed above regarding spatial variation only.

Photosynthesis traits patterns predicted by the optimality theory above can be linked with stem xylem water transportation via stomatal behavior, as given by Fick’s law,

2 $${g}_{s}=\frac{{A}_{{\mathrm{area}}}}{{c}_{a}-{c}_{i}}$$

Where *g*_*s*_ is stomatal conductance (μmol CO_2_ m^−2^ s^−1^), *A*_area_ is CO_2_ assimilation rate per leaf area (μmol CO_2_ m^−2^ s^−1^), and leaf internal (*c*_i_, ppm) and external (*c*_a_, ppm) CO_2_ concentration.

We focus on daytime conditions that produce maximum rates of transpiration and photosynthesis, when water loss through stomata must equal water transport through xylem, assuming no change of stored water in the xylem on annual scales^8,48^:

3 $$\frac{E}{{A}_{L}}=\frac{1.6\cdot {g}_{s}\cdot {{\mbox{VPD}}}_{{la}}}{{P}_{{\mathrm{atm}}}}={K}_{s}\cdot \Delta {\Psi }_{\max }\cdot \frac{{A}_{S}}{{A}_{L}}\cdot \frac{1}{h}$$

Where *E*/*A*_*L*_ is water transpired per leaf area surface (mol m^−2^ s^−1^), VPD_la_ is leaf-to-air VPD, *P*_atm_ is atmospheric pressure (MPa), *K*_s_ is sapwood-specific hydraulic conductivity (mol m^−1^ s^−1^ MPa^−1^); *A*_*S*_/*A*_*L*_ is the ratio of sapwood to leaf area (m^2^ m^–2^), ΔΨ_max_ is the maximum decrease in water potential from soil to leaves (MPa), and h is the transpiration stream path length (m), roughly equivalent to plant height, 1.6 × *g*_s_ × VPD_la_/*P*_atm_ denotes “water loss through stomata,” and *K*_s_ ΔΨ_max_ *A*_*S*_/*A*_*L*_/*h* denotes water transport through xylem.

Combining the above two equations we obtain a link between water transportation and photosynthesis (Eq. 1).

Equation (1)could be rearranged to focus on carbon gain:

4 $${A}_{{\mathrm{area}}}=\frac{{K}_{s}\cdot \Delta {\Psi }_{\max }\cdot \frac{{A}_{S}}{{A}_{L}}}{h\cdot 1.6\cdot {{\mbox{VPD}}}_{{la}}}\cdot \left({c}_{a}-{c}_{i}\right)\cdot {P}_{{\mathrm{atm}}}$$

Note that Eq. (1) was presented on whole-tree level, but was tested using shoot level traits^8^, as well as in this study. Here we disregard diurnal or seasonal variation. Relationships could be very different at other time scales^10^.

Compared to wet sites, drier sites, despite smaller *g*_*s*_, should inevitably have a larger *E*/*A*_*L*_^85^ and more negative ΔΨ_max_^36^ due to higher VPD. Therefore plants in drier sites should have smaller maximum tree height (Eq. 3), and more negative turgor loss point (TLP, Mpa) to increase hydraulic resistance (note that TLP must be more negative than ΔΨ_max_)^86,87^. Equation (3) implies that in drier sites with high VPD, plants require a larger *A*_*S*_/*A*_*L*_ and/or larger *K*_*s*_ in order to support the same amount of photosynthesis with enhanced transpiration. Following the xylem safety-efficiency trade-off^88–91^, plants at drier sites would be expected to have lower hydraulic conductivity (*K*_*S*_). Although arguments against this trade-off exist^32,35,89^, here we present testable hypotheses expected by the trade-off. At dry sites, lower hydraulic conductivity is often associated with smaller vessel lumen diameter, higher vessel density, and higher wood density^39–41^. Such patterns have been observed along an Australian rainfall gradient^36,92^, but no effect of rainfall on vessel lumen diameter was reported elsewhere^93,94^. Plants in drier sites should have increased hydraulic safety—more negative TLP and more negative P50 (water potential at 50% loss of conductivity)^36,95–99^. In short, toward drier sites, we would expect to see higher *A*_*S*_/*A*_*L*_ and more negative TLP. The safety-efficiency trade-off implies lower *K*_*S*_, smaller vessel lumen diameter, higher vessel density, and higher wood density.

The trade-off between K_*s*_ and *A*_*S*_/*A*_*L*_ is also embedded in the variance of traits in Eq. (3). *K*_*S*_ and *A*_*S*_/*A*_*L*_ could vary by two orders of magnitude (100-fold variation)^37^ on a global scale, while *c*_*i*_/*c*_*a*_ and *A*_area_ vary much less (*c*_*i*_/*c*_*a*_: 2-fold; *A*_area_: 10-fold)^9,100^. This leads to a trade-off between *K*_*S*_ and *A*_*S*_/*A*_*L*_ (i.e., *K*_*S*_ × *A*_*S*_/*A*_*L*_ should vary less than either of them). However, given that there are also variations of *c*_*i*_/*c*_*a*_, *A*_area_, *h*, and ΔΨ_max_, it is possible that different species range along a spectrum from high *A*_area_ and *E*/*A*_*L*_ to low *A*_area_ and *E*/*A*_*L*_ while always satisfying Eq. 3^7^.

In short, the above review leads to hypotheses that plants in drier (normally also brighter) sites tend to develop a photosynthesis mechanism with less stomatal conductance and lower *c*_*i*_, stronger photosynthetic capacities (larger *V*_cmax25_, *J*_max25_, and *A*_area_) with more maintenance cost (higher *R*_*d*_) and larger transpiration per leaf area which the water transport system would adjust to with higher *A*_*S*_/*A*_*L*_, lower Ks, lower tree height and more negative TLP. We break the above prediction down into 14 testable hypotheses (Table 1) and test each of them along a typical tropical VPD gradient in West Africa.

### Reporting summary

Further information on research design is available in the Nature Portfolio Reporting Summary linked to this article.

## Supplementary information

Supplementary information

Reporting summary

## Data Availability

Raw data behind this manuscript could be found in this repository: 10.6084/m9.figshare.29230625^101^. Leaf-level trait data are also available through the TRY trait database. For further information or specific data requests, please contact the corresponding author.

## Code availability

The code used for data processing, analysis, and figure reproduction is available in this repository: 10.6084/m9.figshare.29230625. This repository includes all relevant R code and scripts necessary to reproduce the figures and analysis presented in the manuscript. There are no restrictions on access to the code. Users are encouraged to utilize and adapt the code for further research, with proper citation of the original work.
