# Supplementary material for: A test of ecophysiological theories on tropical forest functional traits along a VPD gradient
Source: Commun Biol. 2025 Jul 9;8:1031. doi: 10.1038/s42003-025-08420-1 (PMC12241516; doi:10.1038/s42003-025-08420-1)
Supplement: Supplementary file 1 — Supplementary information [file 42003_2025_8420_MOESM1_ESM.pdf]

Supplementary for

"A test of ecophysiological theories on tropical forest functional traits along a VPD gradient"

Huanyuan Zhang-Zheng[1], Yadvinder Malhi[1], Kasia Ziemińska[1], Agne Gvozdevaite[1], Theresa Peprah[2], Mickey Boakye[3], Stephen Adu-Bredu[2], Jesús Aguirre-Gutiérrez[1], Sam Moore[1], David Sandoval[4], Minxue Tang[4], Iain Colin Prentice[4]*, Imma Oliveras Menor[1 5]**

Affiliations:

1. Environmental Change Institute, School of Geography and the Environment, University of Oxford, Oxford, United Kingdom
2. Forestry Research Institute of Ghana, Council for Scientific and Industrial Research, Kumasi, Ghana
3. Department of Environmental Science, Policy, and Management, University of California, Berkeley, CA, 94720, USA
4. Georgina Mace Centre for the Living Planet, Department of Life Sciences, Imperial College London, Silwood Park Campus, Buckhurst Road, Ascot, SL5 7PY, UK
5. AMAP (Botanique et Modelisation de l'Architecture des Plantes et des Végétations), Université de Montpellier, IRD, CIRAD, CNRS, INRAE, Montpellier, France

* Corresponding authors

Supplementary figures and tables

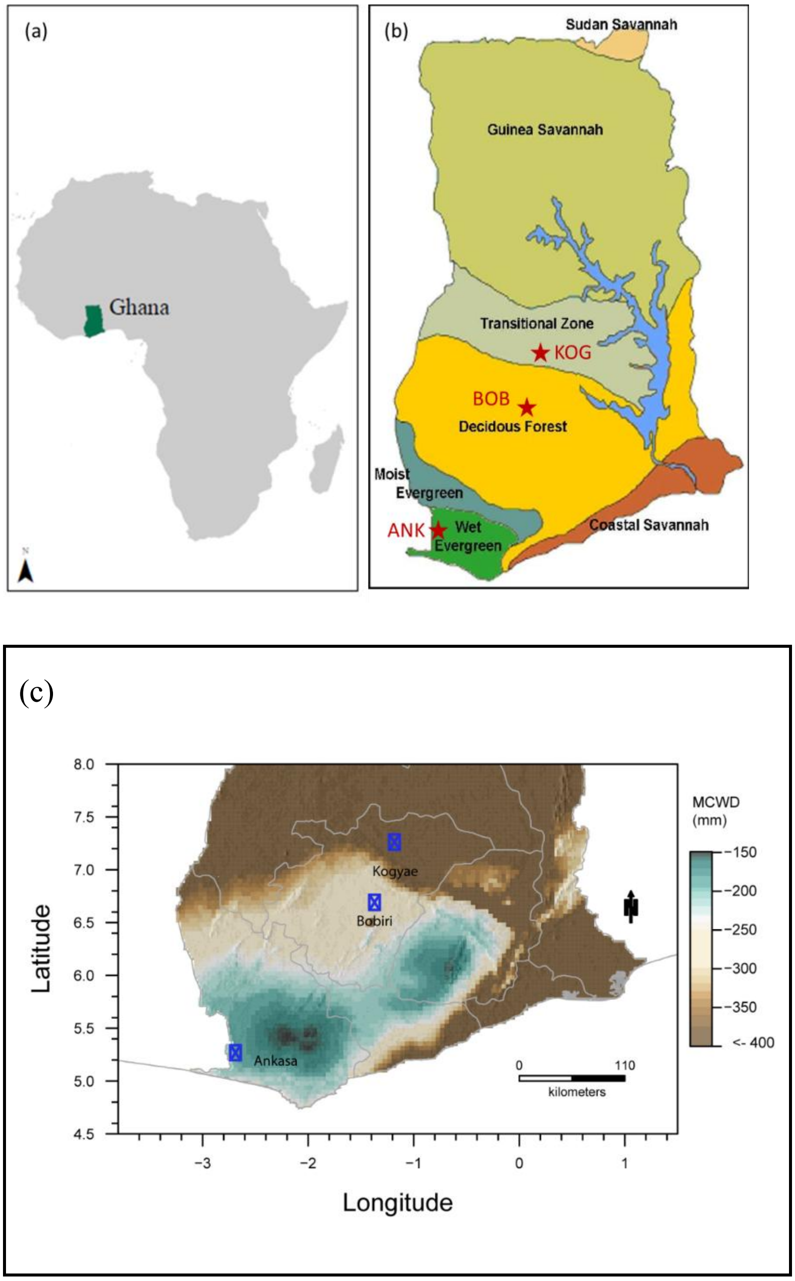

Figure S1 Map of the location of (a) Ghana within the African continent (b) and the study sites and forest types in Ghana [1]. (c) showed study sites over a map of maximum climate water deficit (MCWD) [2].

Table S1 Summary information of plot characteristics

		Wet >> Dry						
Plot		ANK-01	ANK03	BOB-01	BOB-02	KOG02	KOG-04	KOG05
Lat		5.27	5.27	6.7	6.69	7.26	7.3	7.3
Long		-2.69	-2.69	-1.32	-1.34	-1.15	-1.18	-1.16
Elev	(m)	114	86	277	281	229	230	221
PPFD	(mol /m2/day)	30.97	30.97	35.58	35.58	44.26	44.26	44.26
MAP	(mm)	2050	2050	1500	1500	1200	1200	1200
VPD	(kPa)	0.284	0.284	0.534	0.534	0.722	0.722	0.722
MAT	(°C)	25	25	25.7	25.7	26.4	26.4	26.4
MCWD	(mm/yr)	-13	-13	-374	-374	-412	-412	-412
Vegetation type		LR	SLR	SemR	SemR	SSF	STF	WS
BA	(m2/ha)	28	25.8	22.9	31.1	17.5	13.5	12.4
Ind	(#/ha)	59	31	27	41	36	33	22
Trees	(#/ha)	477	414	745	486	157	190	145
Sp/Gen/Fam		35/29/22	13/13/9	16/13/8	14/13/8	15/14/10	11/11/7	7/6/5
P	(mg/kg)	146.8	109.7	77.8	258.3	67.2	74.6	81.9
N	(%)	0.17	0.12	0.09	0.16	0.06	0.05	0.04
C	(%)	2.61	1.91	0.8	1.71	0.72	0.67	0.62
Ca	(mg/kg)	26.8	40	306.3	657.6	378.9	308	237.1
K	(mg/kg)	32.3	33.7	47.6	49	42.5	35.6	28.7
Mg	(mg/kg)	42	29.2	79.7	133.7	75.6	78.7	81.3
Sand	(%)	63.1	75.9	64.2	46.7	82.4	79.7	76.9
Clay	(%)	21.6	12.8	6.7	28.8	2.3	3.3	4.3

Note 1: Plot coordinates (Latitude[Lat]/Longitude[Long]) are provided in degrees. Elev, elevation; MAP, mean annual precipitation; VPD, mean annual vapour pressure deficit; PPFD, photosynthetic photon flux density, calculated from shortwave radiation *0.5; MAT, mean annual temperature; MCWD, Maximum Cumulative Water Deficit; Vegetation type (LR, lowland rainforest; SLR, semi-flooded lowland rainforest; SemR, semi-deciduous rainforest; SSF, seasonal semi-deciduous forest; STF, seasonal transitional rainforest; WS, woodland savanna); BA, total basal area in a plot; Ind, number of tree individuals sampled; Trees, total number of trees larger than 10cm diameter at breast height; Soil nutrients (P, phosphorus; N, nitrogen; C, carbon; Ca, calcium; Mg, magnesium), and soil percentage of sand (Sand) and of clay (Clay). Data from [3,4]

Note 2: the swampy rainforest (ANK03) is partly inundated during the wet season but not ANK01, which is located on a hill and never inundated. From KOG02 (dry forest), KOG04 to KOG05 (savanna), forest plots become more deciduous with a smaller number of trees.

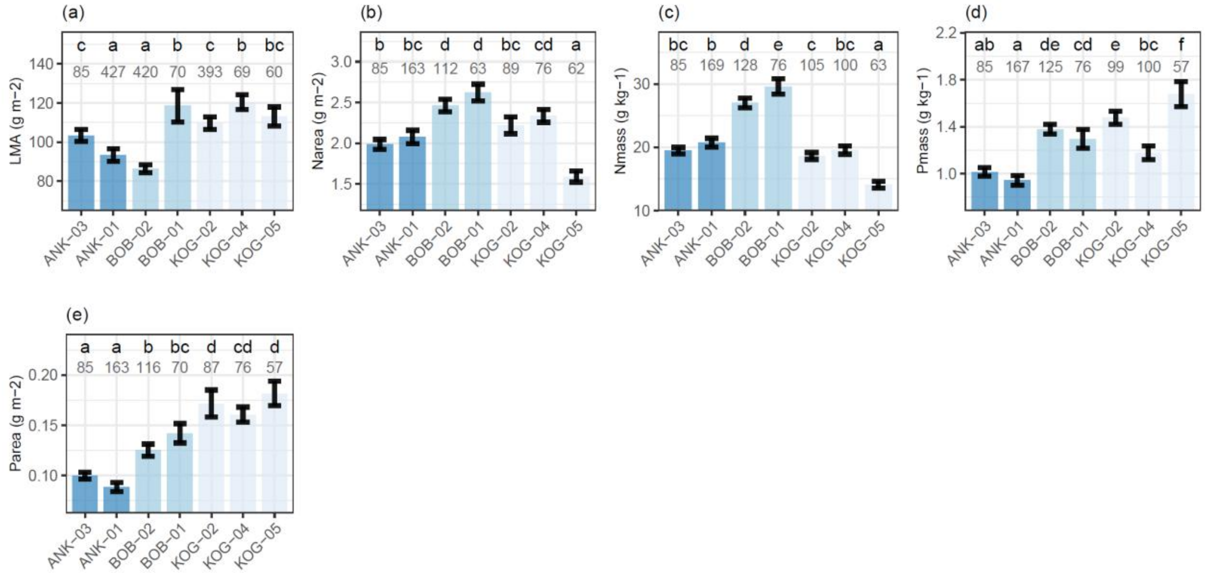

Figure S2 Community weighted mean (with standard error) of variables associated with leaf economy from wet to dry plots (left to right). Plots were ordered from left to right according to vapour pressure deficit (VPD). The number denotes the number of samples, which could be a leaf, a branch or a tree etc. The letters denote significance (P<0.05) in plot-to-plot difference. Data reported in this study are slightly different to the these two studies about the same site [4,5] because there are more sampling in this study. Samples were taken each month from October 2014 to September 2016. Data here are provided for completeness and for future researchers 'convenience. The figure show (a) leaf mass per area (g m^{-2}) (b) leaf nitrogen content per leaf area (g m^{-2}) (c) leaf nitrogen content per leaf mass (g kg^{-1}) (d) leaf phosphorus content per leaf mass (g kg^{-1}) and (e) leaf phosphorus content per leaf area (g m^{-2}). Although data distributions are not shown in these panels, they are presented in Figure S20.

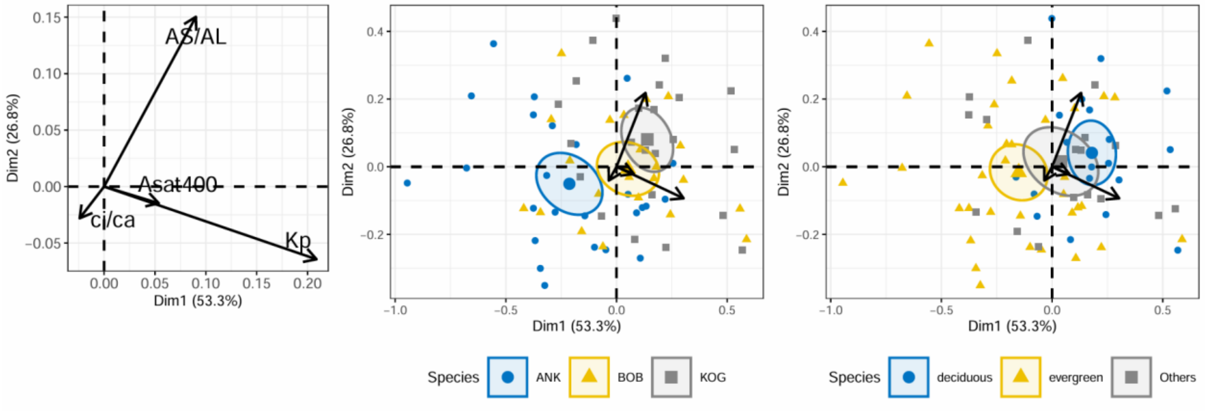

Figure S3 Principal components analysis for A_S/A_L , ci/ca, Asat400 and Kp. Similar to Figure 5 but replacing Vcmax25 with Asat400.

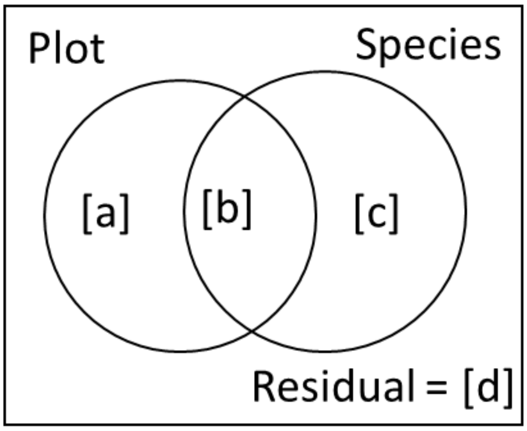

[a] Variance incurred by changing plot for the same species

[b] Variance incurred by changing plot and changing species

[c] Variance incurred by changing Species but not plot (inter-specific variance in a plot)

[d] Variance incurred by anything else but not changing plot and not changing species. It includes intra-specific variation (e.g., changing measurement leaves) and measurement error because for most of the traits, [d] exist only when multiple measurements were collected for one species. If the changing species induced variance [c] is larger than [d], it is safe to conclude that the variation of trait (overall in Ghana) is dominated by changing species, instead of intra-specific variation

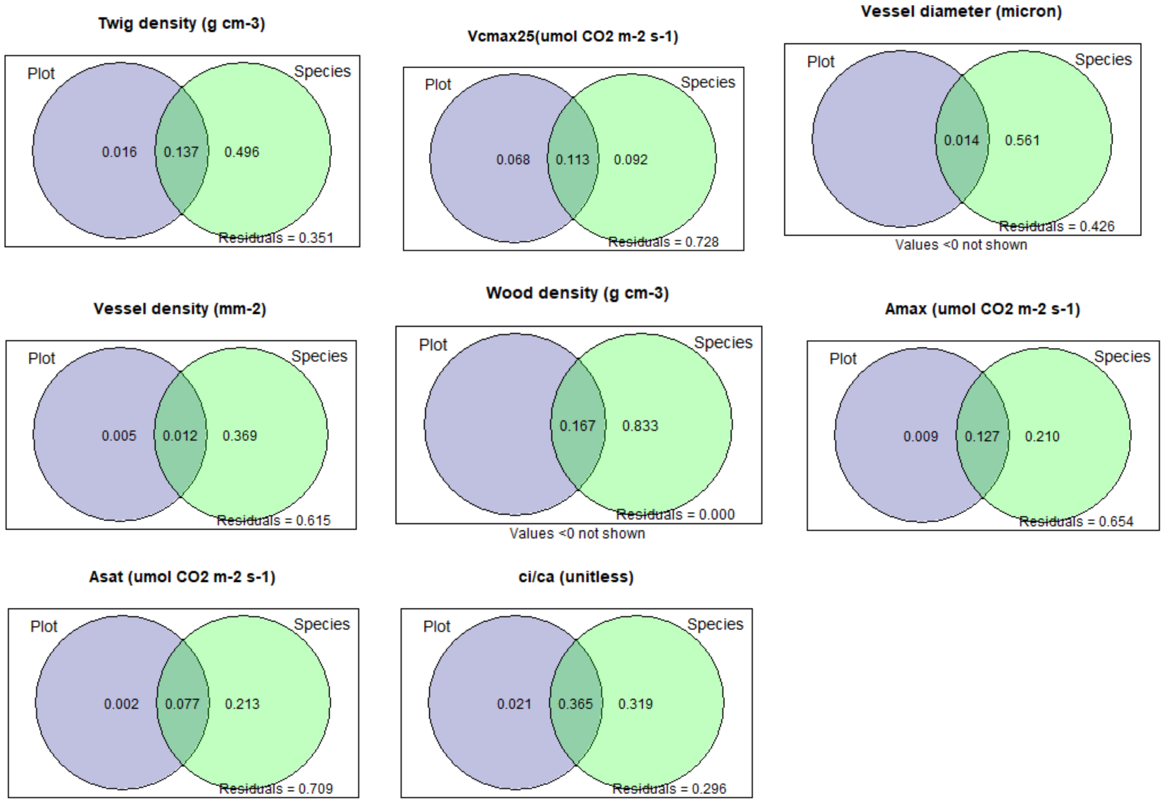

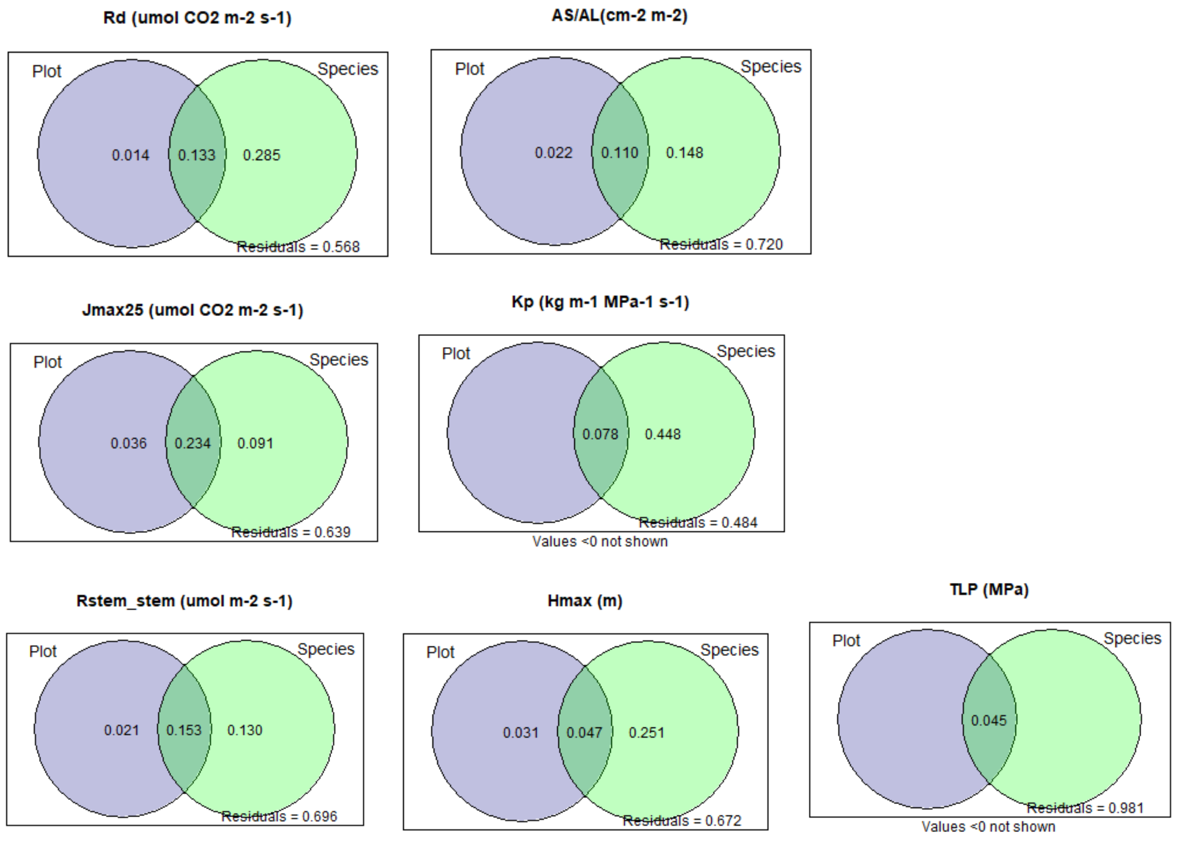

Figure S4 Variance partitioning into plot and species. Please see table 1 for definition of traits. Meanings of each number in the circles are explained in the top panel. Note that Vcmax, LMA Narea and Parea from the same plots were published in [5], and A_{sat400} $A_{sat2000}$ LMA, Nmass and Pmass from the same plots were published in Oliveras et al[4]. Values are not mathematically identical due to (1) different methods of variance partitioning and (2) one more year sampling than the previous publications

Table S2 General Additive Models fitted for each trait, including twig density (g cm^{-3}), wood density (g cm^{-3}), potential specific hydraulic conductivity (Kp, kg m^{-1} Mpa^{-1} s^{-1}), Rubisco carboxylation capacity at 25 C° (V_{cmax25}, umol CO_2 m^{-2} s^{-1}), electron transport capacity at 25 C° (J_{max25}, umol CO_2 m^{-2} s^{-1}), turgor loss point (TLP, MPa), Sapwood to leaf area ratio (Huber value) (AS/AL, cm2 m^{-2}), light saturated assimilation rate at 2000 ppm Asat2000 (umol CO2 m^{-2} s^{-1}), light saturated assimilation rate at 400 ppm Asat400 (umol CO2 m^{-2} s^{-1}), leaf dark respiration (Rd, umol CO2 m^{-2} s^{-1}), the ratio between leaf-internal and ambient CO2 (ci/ca, unitless), plant stature, calculated as maximum tree height of a species in the study plot (Hmax, m), vessel density (mm^{-2}), and vessel lumen diameter (μm).V_{cmax25}, J_{max25}, and TLP are shown with SITE only because these traits are not studied at plot sclae.

Trait	Explanatory variables	Estimate	Ref.df	F	p-value	Rsquare	Deviance explained	edf
Twig Density	**s(SITE)**	0.57	2	**63.223**	**0.00**	0.039	0.041	1.773
	s(PLOT)	0.57	6	0.486	0.11	0.039	0.041	0.645
	s(SITE,PLOT)	0.57	6	2.160	0.05	0.039	0.041	1.559
Wood Density	s(SITE)	0.59	2	2060.470	0.18	0.167	0.170	1.045
	s(PLOT)	0.59	6	2.262	0.60	0.167	0.170	0.144
	s(SITE,PLOT)	0.59	6	588.129	0.20	0.167	0.170	4.713
Hydraulic conductivity	**s(SITE)**	53.16	2	**5.807**	**0.00**	0.046	0.054	1.607
	s(PLOT)	53.16	6	0.045	0.30	0.046	0.054	0.213
	s(SITE,PLOT)	53.16	6	0.047	0.30	0.046	0.054	0.220
Vcmax25	**s(SITE)**	29.06	2	**5.282**	**0.00**	0.066	0.076	1.682
Jmax25	**s(SITE)**	54.88	2	**7.156**	**0.00**	0.087	0.098	1.755
Turgor	**s(SITE)**	0.17	2	**5.709**	**0.00**	0.044	0.051	1.726
AS/AL	**s(SITE)**	6.89	2	**15.935**	**0.00**	0.089	0.098	1.815
	s(PLOT)	6.89	6	0.074	0.44	0.089	0.098	0.392
	s(SITE,PLOT)	6.89	6	0.102	0.43	0.089	0.098	0.517
Asat2000	s(SITE)	19.49	2	37.549	0.07	0.136	0.140	0.311
	s(PLOT)	19.49	6	<0.01	0.15	0.136	0.140	0.000
	**s(SITE,PLOT)**	19.49	6	**105.153**	**0.00**	0.136	0.140	5.106
Asat400	**s(SITE)**	6.66	2	**390.344**	**0.02**	0.078	0.082	1.486
	s(PLOT)	6.66	6	3.453	0.46	0.078	0.082	0.732
	s(SITE,PLOT)	6.66	6	36.482	0.23	0.078	0.082	2.949
Dark Respiration	**s(SITE)**	1.89	2	**3083.951**	**0.02**	0.148	0.151	1.643
	s(PLOT)	1.89	6	3.899	0.36	0.148	0.151	0.228
	**s(SITE,PLOT)**	1.89	6	**615.870**	**0.04**	0.148	0.151	3.782
ci/ca	**s(SITE)**	0.79	2	**75.174**	**0.00**	0.387	0.393	1.969
	s(PLOT)	0.79	6	<0.01	0.50	0.387	0.393	0.000
	s(SITE,PLOT)	0.79	6	<0.01	0.50	0.387	0.393	0.000
Vessel Diameter	s(SITE)	81.08	2	<0.01	0.83	0.016	0.024	0.000
	s(PLOT)	81.08	6	0.140	0.24	0.016	0.024	0.564
	s(SITE,PLOT)	81.08	6	0.442	0.13	0.016	0.024	1.427
Vessel Density	s(SITE)	50.18	2	1.743	0.07	0.014	0.019	1.272
	s(PLOT)	50.18	7	<0.01	0.75	0.014	0.019	0.000
	s(SITE,PLOT)	50.18	7	<0.01	0.75	0.014	0.019	0.000

Models Explanation: For each trait, a formula is fitted as trait ~ s(SITE, bs = "re") + s(PLOT, bs = "re") + s(SITE, PLOT, bs = "re"). The table shows estimated degree of freedom (edf), reference degree of freedom (Red.df), F statistic, p-value, components of the variance explained, name of the trait, Adjusted R square and Deviance explained by the whole model. Although p-value is given, readers are suggested to focus on F value. A relatively large F value at s(SITE) signals most variance owned by site-to-site variation. A relatively large F value at s(PLOT) signals most variance owned by plot-to-plot variation. A relatively large F value at s(SITE, PLOT) signals that after acknowledging for site-by-site variation, there is a large plot-to-plot variation within each site. s(PLOT) appears insignificant for all traits because the variance is taken away by s(SITE) and s(SITE, PLOT). If we fit trait ~ s(PLOT, bs = "re"), without SITE, the s(PLOT) would become significant, which has been told by ANOVA in the main text.

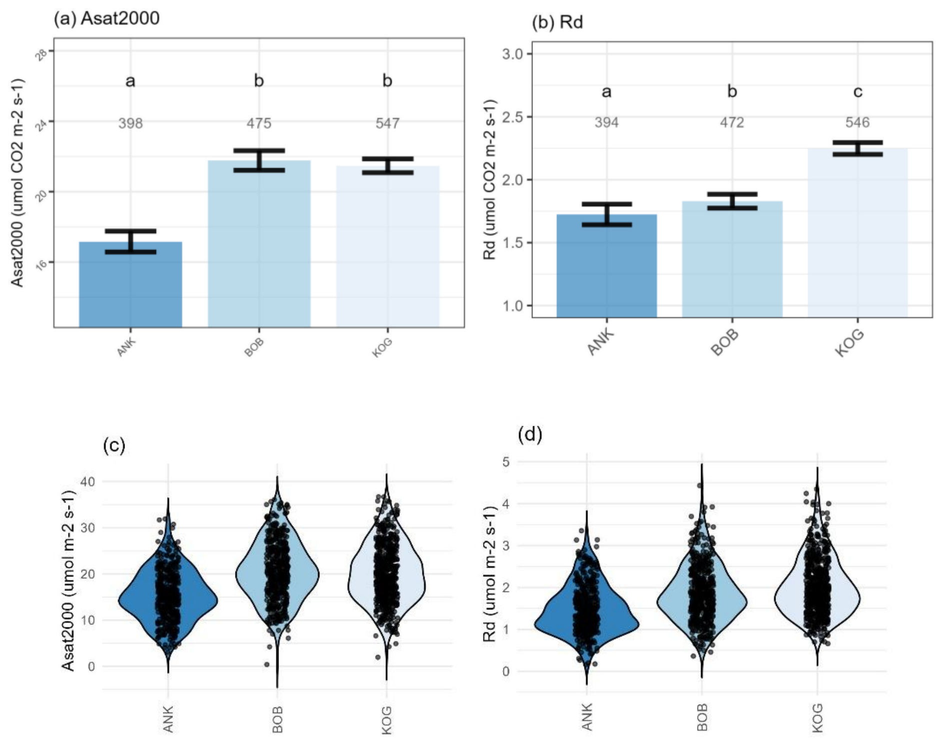

Figure S5 Site scale community weighted mean of measured traits (with standard error) for (a) light saturated assimilation rate at 2000 ppm $A_{sat2000}$ (umol CO_2 m^{-2} s^{-1}), (b) leaf dark respiration (Rd, umol CO_2 m^{-2} s^{-1}). The figure shows study plots from the wettest (left) to the driest (right) plot. Forest plots are arrayed from left to right in order of VPD. The number denotes the number of samples, which could be a leaf, a branch, a tree or a species depending on the variable. The letters denote significance (ANOVA, $P<0.05$) in site-to-site difference. This figure is equivalent to Figure 3 but on the site scale. $A_{sat2000}$ and Rd are shown on the site scale because the nested factor of these two traits appear significant in General additive models (Table S2). (c) and (d) display the data distributions behind (a) and (b).

Supplementary note 1

Supplementary note 1 provides a detailed report about hypothesis 14.

We hypothesized that the product of K_P (specific xylem hydraulic conductivity) and A_S/A_L (sapwood to leaf area) vary less than K_P or A_S/A_L themselves, and there is a trade-off (negative correlation) between K_P and AS/AL (Supplementary Method). As the trade-off between Ks (well associated with K_P) and A_S/A_L has been observed on a global scale [6], here we also plot K_P versus A_S/A_L for readers' convenience in comparison with measurements from Ghana VPD gradient. We estimated K_P for species reported in [6] by collecting vessel lumen diameter and

vessel density from XFT database [7], with the same calculation method as K_P of Ghana VPD gradient.

For Ghana, both hypotheses were rejected, as we see a positive correlation between K_P and A_S/A_L (slope = 0.95, R-squared : 0.0598, P-value : 0.0224) and the coefficient of variance is found largest for K_P * A_S/A_L.

For a global dataset [6], there is a negative correlation between K_P and A_S/A_L (slope =-0.638, R-squared: 0.153, P-value : <0.001) which agreed with the hypothesis but the coefficient of variance of K_P * A_S/A_L is still larger than that of either K_P or A_S/A_L.

Therefore, hypothesis 14 in Table 1 is rejected in this study. The negative correlation between K_P and A_S/A_L emerge on global scale probably because of confounding effect with other environmental variables. The different patterns emerged at different scale could also result from a Simpson's paradox. For example, the drier sites (KOG) have higher K_P , higher twig density and higher wood density than the wetter sites on site scale (Figure 4), but we also found K_P negatively correlated with twig density on species scale (Figure S3)

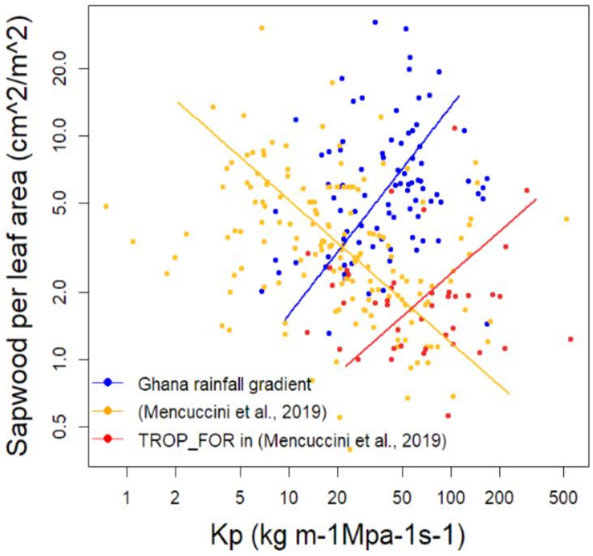

Figure S6 The correlation between sapwood to leaf area (AS/AL) and potential sapwood hydraulic conductivity (Kp) for Ghana VPD gradient (ANK, BOB and KOG all together) and species included in [6]. The figure was drawn on species scale (one scatter point is one species).

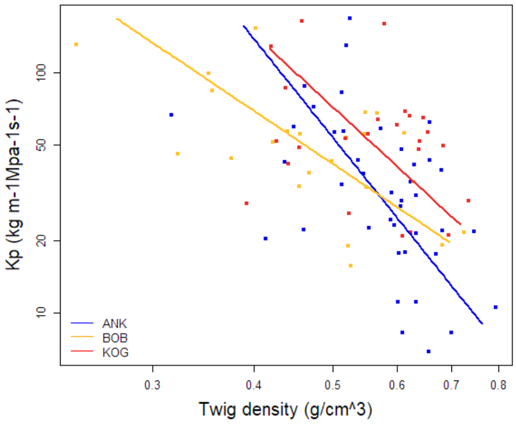

Figure S7 The correlation between twig density (g/cm3) and potential sapwood hydraulic conductivity (Kp) for site ANK, BOB and KOG. The figure was drawn on species scale (one scatter point is one species).

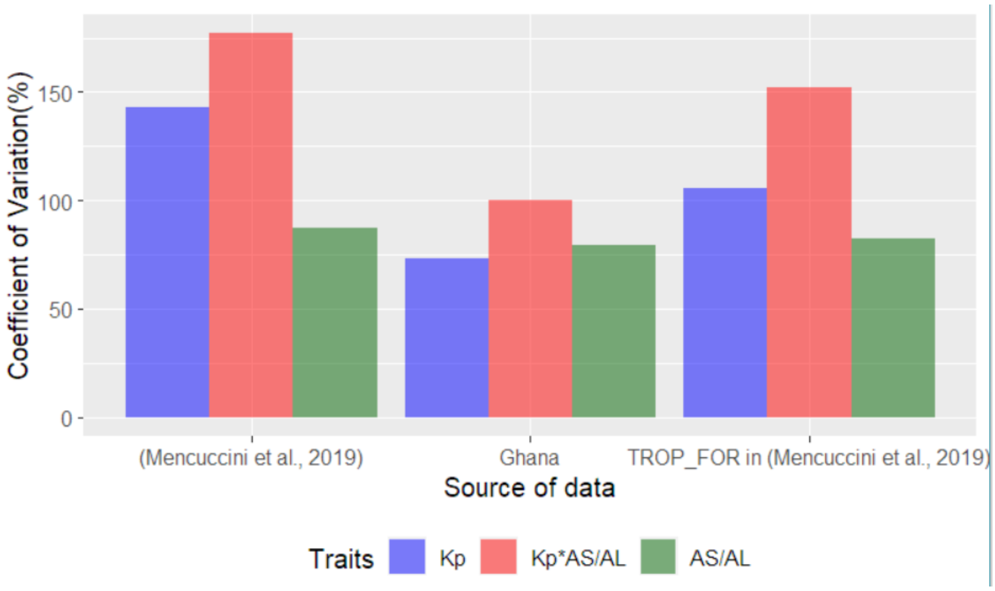

Figure S8 Coefficient of variation (%) for data points shown in figure S6, potential sapwood hydraulic conductivity (Kp), sapwood area to leaf area (AS/AL) and the product of Kp and AS/AL

Field photos

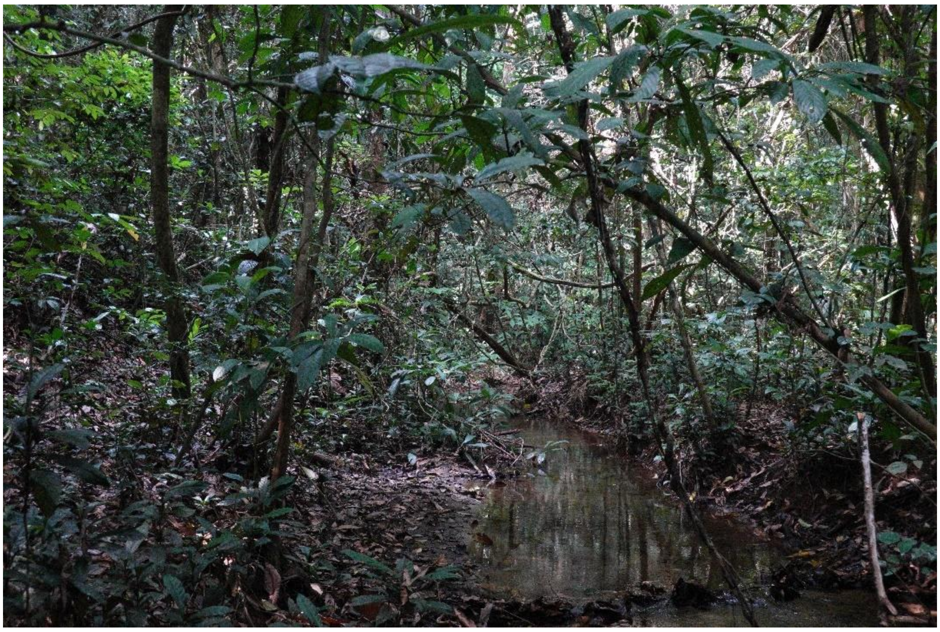

Figure S9 Field photo of study plot ANK03. There is a stream running through ANK03 which largely floods the plot in the wet season. Ankasa - ANK03. Photo Credit: Huanyuan Zhang-Zheng taken in January 2022. This image has been reproduced from a previous publication[8].

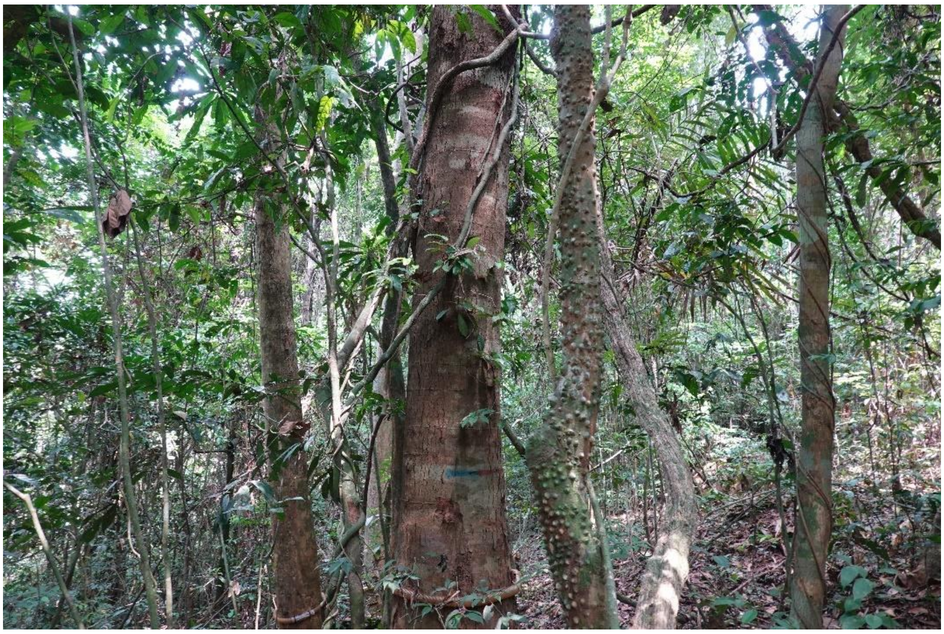

Figure S10 Field photo of study plot ANK01 or ANK02 (we could not tell which one it belongs to). They are located on well-drained local hilltops. The Flux Tower GH-Ank is situated next to ANK01 and ANK02. These two plots are very similar to each other. Photo Credit: Huanyuan Zhang-Zheng taken in January 2022. This image has been reproduced from a previous publication[8].

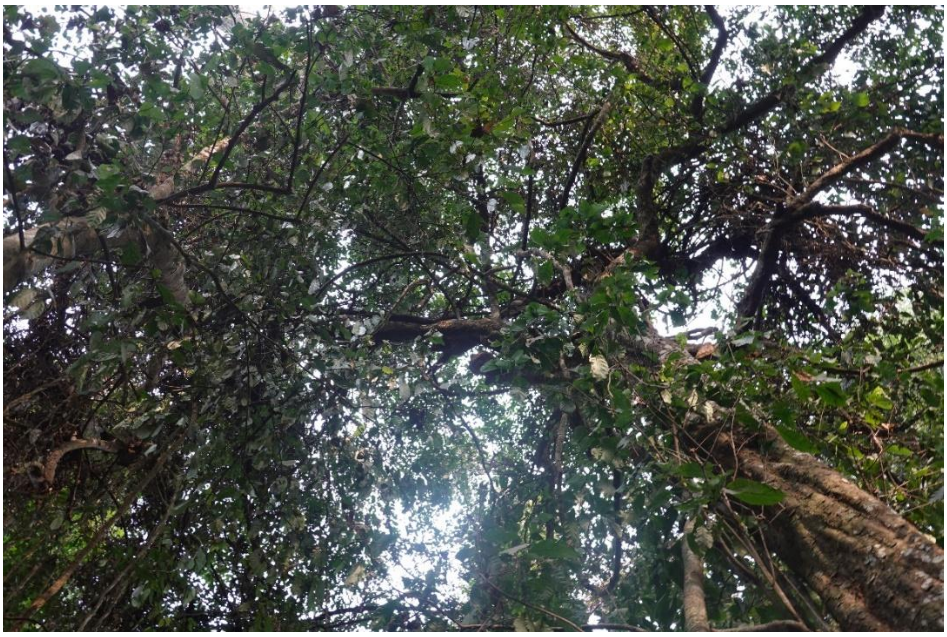

Figure S11 Study plot BOB01. Photo Credit: Huanyuan Zhang-Zheng taken in January 2022. This image has been reproduced from a previous publication[8].

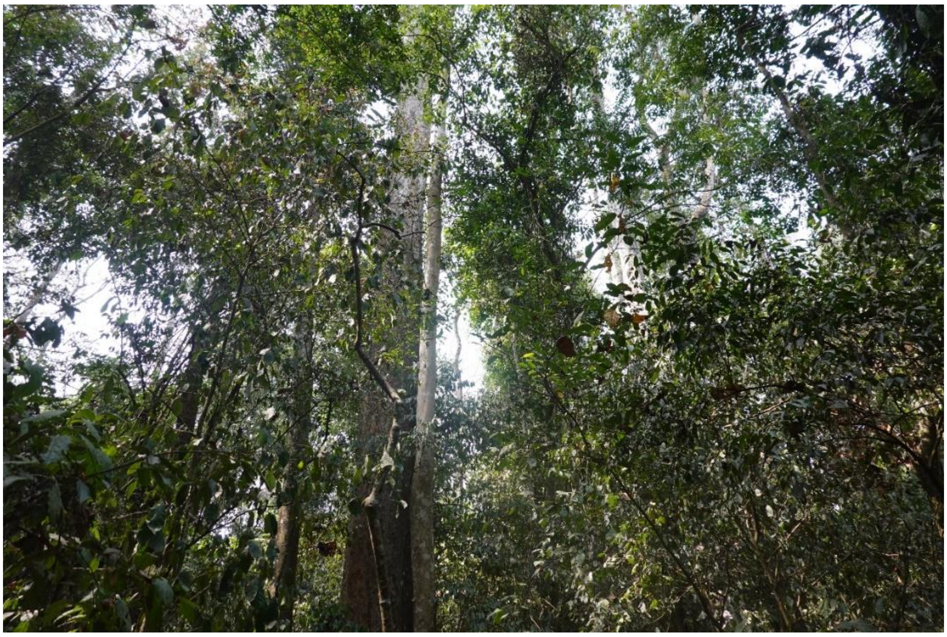

Figure S12 Study plot BOB01. Photo Credit: Huanyuan Zhang-Zheng taken in January 2022. This image has been reproduced from a previous publication[8].

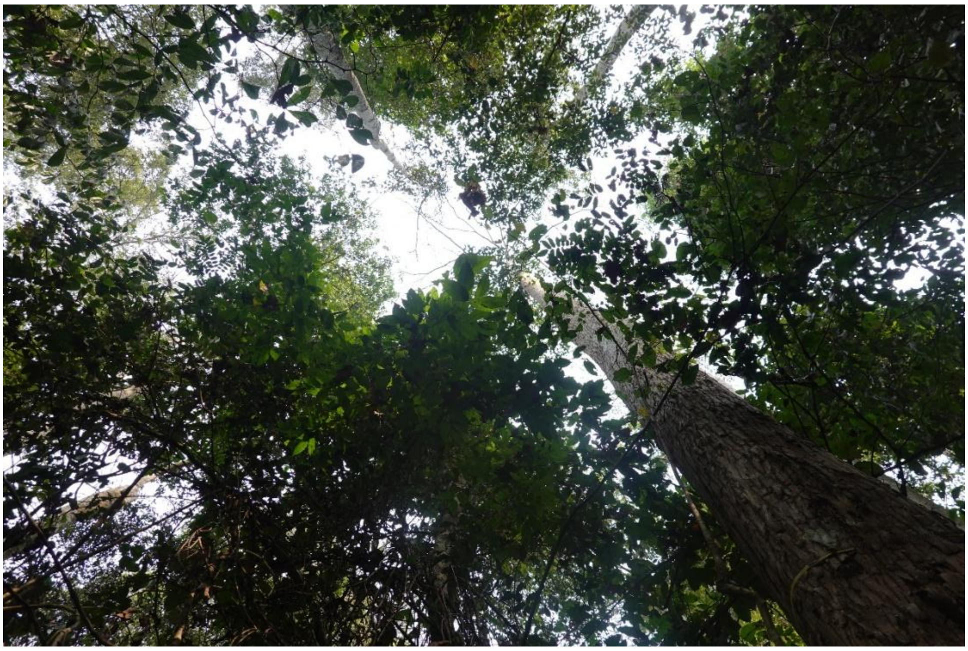

Figure S13 Study plot BOB02. Study plots BOB01 and BOB02 are about 1km apart, both belong to Bobiri forest reserve. Photo Credit: Huanyuan Zhang-Zheng taken in January 2022. This image has been reproduced from a previous publication[8].

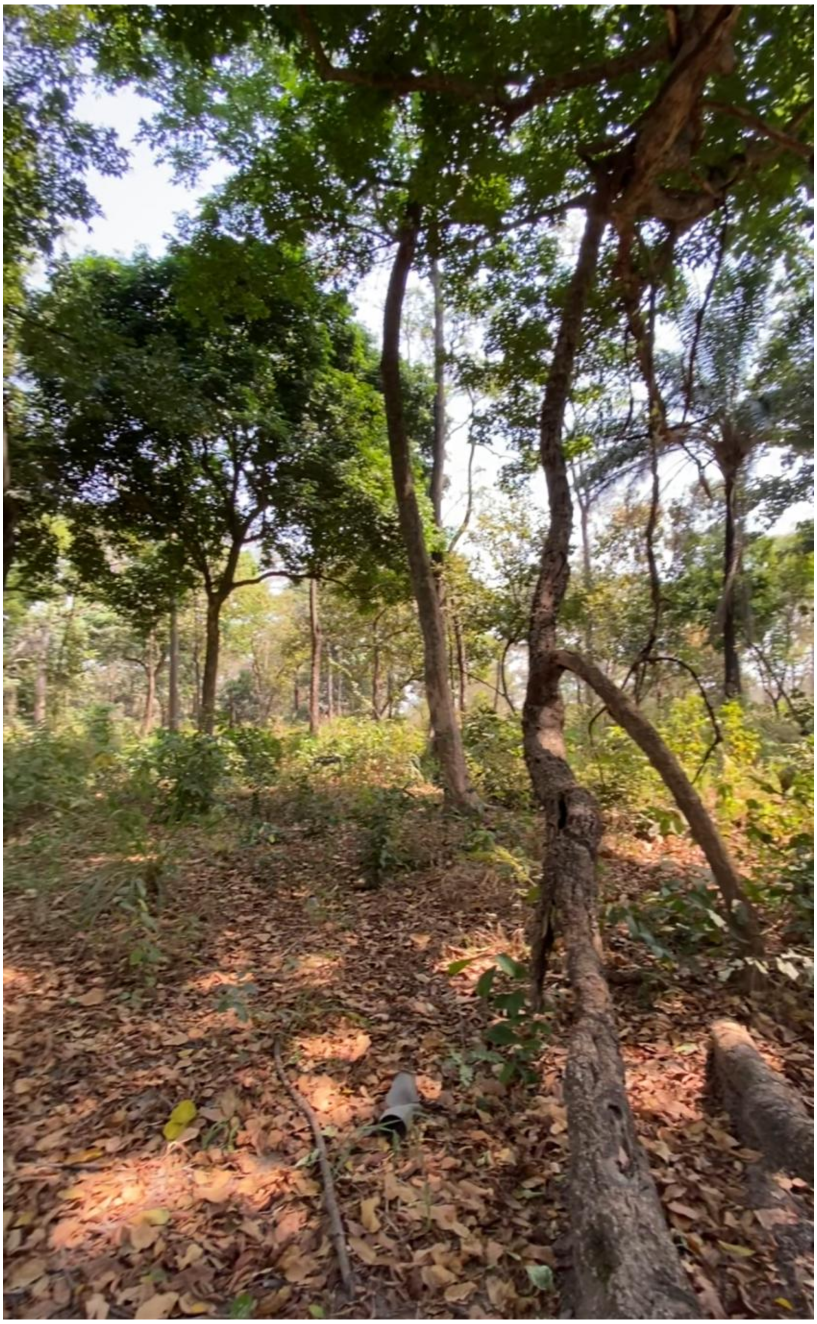

Figure S14 Study plot KOG02. This plot is recognized as dry forests because it has little grass beneath the canopy. The canopy retains leaves all year round. Photo Credit: taken by Huanyuan Zhang-Zheng in January 2022. This image has been reproduced from a previous publication[8].

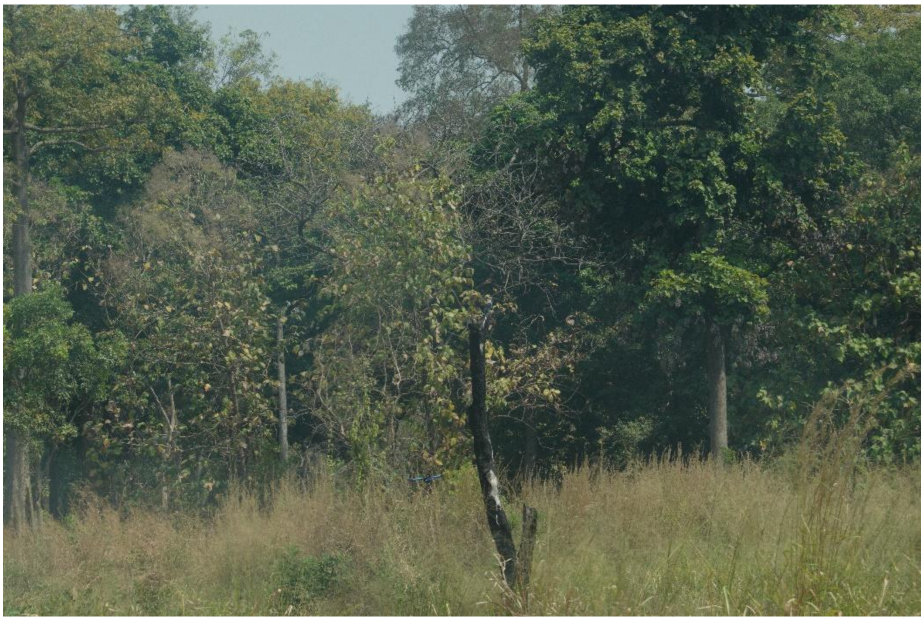

Figure S15 Outside study plot KOG04. This is at the forest-savanna transition. It looks pretty dense from outside. However, inside the plot, there are patches of grass. Photo Credit: taken by Huanyuan Zhang-Zheng in January 2022. This image has been reproduced from a previous publication[8].

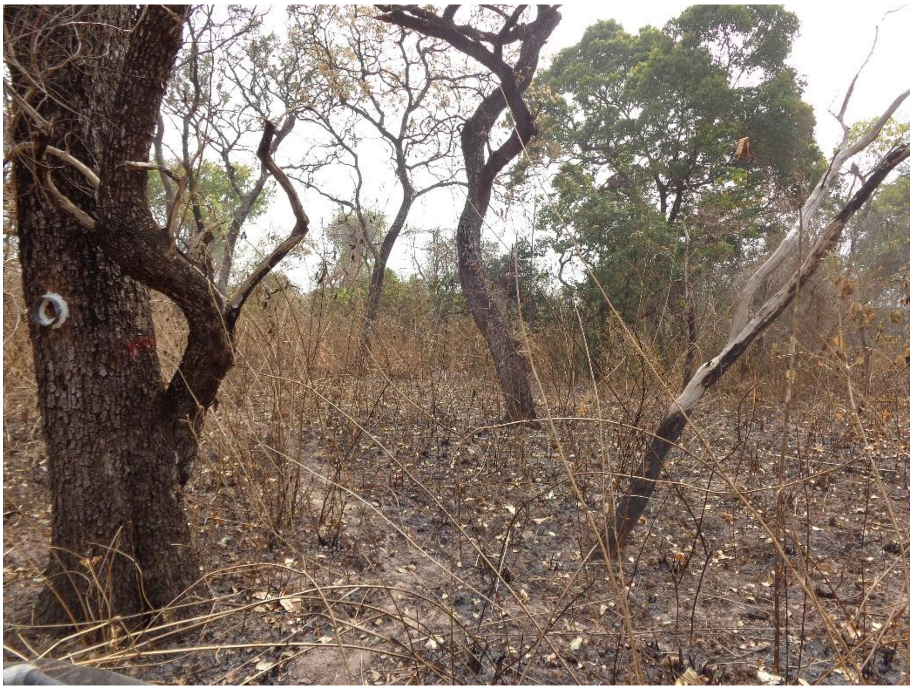

Figure S16 Study plot KOG04. This plot rarely burns (as told by locals), but it looks like this when it does burn. Photo Credit: the photo was shared by Akwasi Duah-Gyamfi. The photo was taken on 03 February 2014. This image has been reproduced from a previous publication[8].

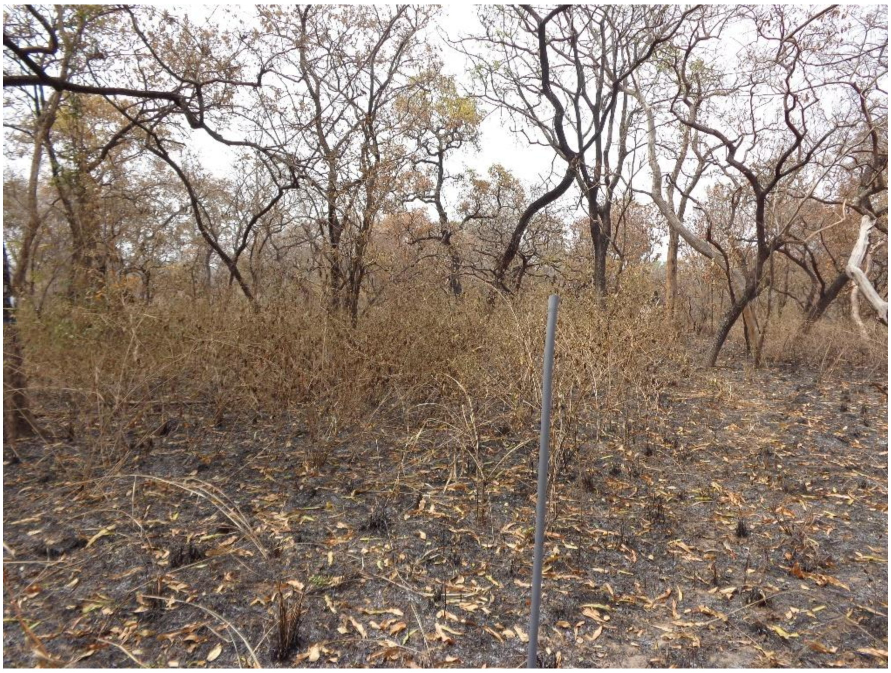

Figure S17 Study plot KOG05. This plot frequently burns. Photo Credit: the photo was shared by Akwasi Duah-Gyamfi. The photo was taken on 06 February 2014. This image has been reproduced from a previous publication[8].

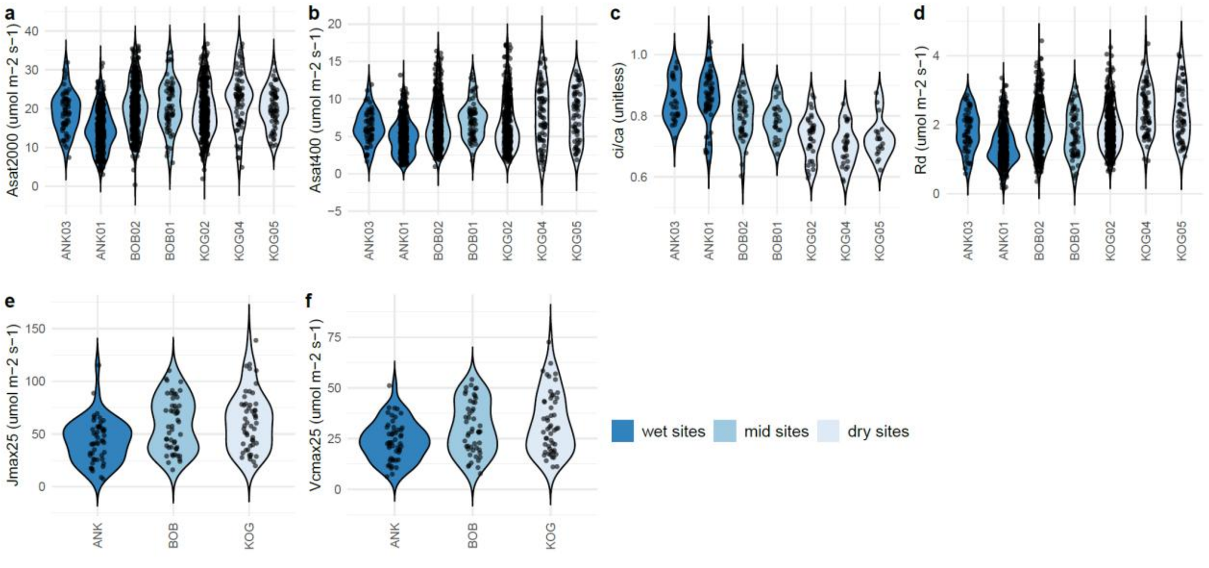

Figure S18 Same as Figure 3 but display the data distribution. The figure shows (a) light saturated assimilation rate at 2000 ppm $A_{sat2000}$ (umol CO_2 m^{-2} s^{-1}), (b) light saturated assimilation rate at 400 ppm A_{sat400} (umol CO_2 m^{-2} s^{-1}), (c) the ratio between leaf-internal and ambient CO_2 (ci/ca, unitless), (d) leaf dark respiration (Rd, umol CO_2 m^{-2} s^{-1}), (e) electron

transport capacity at 25 C° (J_{max25}, umol CO_2 m^{-2} s^{-1}) and (f) Rubisco carboxylation capacity at 25 C° (Vc_{max25}, umol CO_2 m^{-2} s^{-1}).

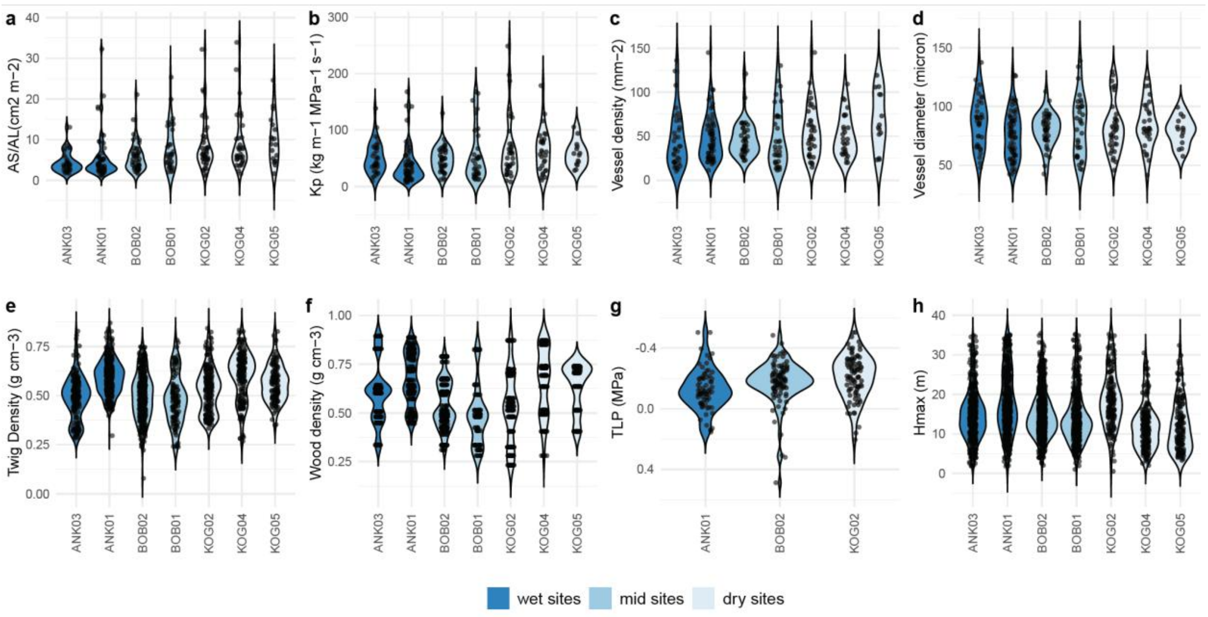

Figure S19 Sane as Figure 4 but display the data distribution. *The figure shows (a)* Sapwood to leaf area ratio (Huber value) (A_S/A_L, cm^2 m^{-2}) (b) potential specific hydraulic conductivity (Kp, kg m^{-1} Mpa^{-1} s^{-1}) (c) vessel density (mm^{-2}) (d) vessel lumen diameter (μm) (e) twig density (g cm^{-3}) (f) wood density (g cm^{-3}) (g) turgor loss point (TLP, MPa) (h) plant stature, calculated as maximum tree height of a species in the study plot (H_{max}, m). Statistics are not applied for Hmax because every tree (>10cm DBH) in the plot is measured.

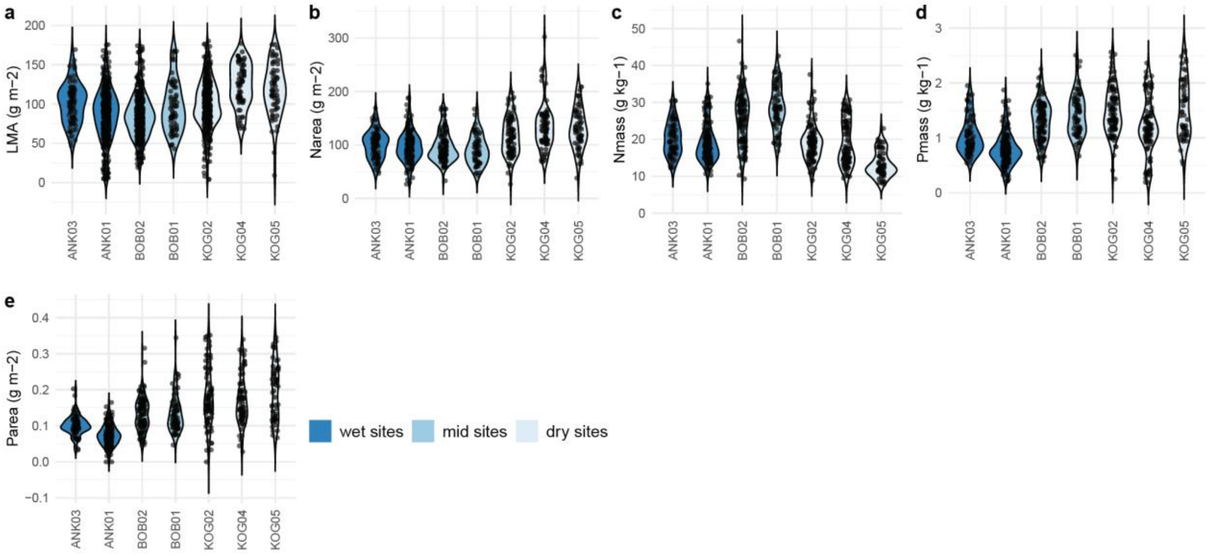

Figure S20 Sane as Figure S2 but display the data distribution. The figure show (a) leaf mass per area (g m^{-2}) (b) leaf nitrogen content per leaf area (g m^{-2}) (c) leaf nitrogen content per leaf

mass (g kg^{-1}) (d) leaf phosphorus content per leaf mass (g kg^{-1}) and (e) leaf phosphorus content per leaf area (g m^{-2})

Supplementary references

1. Appiah, D., Osman, B. & Boafo, J. Land Use and Misuse; Human Appropriation of Land Ecosystems Services in Ghana. *International Journal of Ecosystem* **4**, 24–33 (2014).

2. Aguirre-Gutiérrez, J. *et al.* Drier tropical forests are susceptible to functional changes in response to a long-term drought. *Ecol Lett* **22**, 855–865 (2019).

3. Moore, S. *et al.* Forest biomass, productivity and carbon cycling along a rainfall gradient in West Africa. *Glob Chang Biol* **24**, e496–e510 (2018).

4. Oliveras, I. *et al.* The influence of taxonomy and environment on leaf trait variation along tropical abiotic gradients. *Frontiers in Forests and Global Change* **3**, 18 (2020).

5. Gvozdevaite, A. *et al.* Leaf-level photosynthetic capacity dynamics in relation to soil and foliar nutrients along forest–savanna boundaries in Ghana and Brazil. *Tree Physiol* **38**, 1912–1925 (2018).

6. Mencuccini, M. *et al.* Leaf economics and plant hydraulics drive leaf : wood area ratios. *New Phytologist* **224**, 1544–1556 (2019).

7. Choat, B. *et al.* Global convergence in the vulnerability of forests to drought. *Nature 2012 491:7426* **491**, 752–755 (2012).

8. Zhang-Zheng, H. *et al.* Why models underestimate West African tropical forest primary productivity. *Nat Commun* **15**, 9574 (2024).
