# Supplementary material for: A test of ecophysiological theories on tropical forest functional traits along a VPD gradient
Source: Commun Biol. 2025 Jul 9;8:1031. doi: 10.1038/s42003-025-08420-1 (PMC12241516; doi:10.1038/s42003-025-08420-1)
Supplement: Supplementary file 2 — Reporting summary [file 42003_2025_8420_MOESM2_ESM.pdf]

Corresponding author(s): Prof Iain Colin Prentice; Dr H. Zhang-Zheng; Prof Imma Oliveras-Menor

Last updated by author(s): Jun 5, 2025

Reporting Summary

Nature Portfolio wishes to improve the reproducibility of the work that we publish. This form provides structure for consistency and transparency in reporting. For further information on Nature Portfolio policies, see our Editorial Policies and the Editorial Policy Checklist.

Statistics

For all statistical analyses, confirm that the following items are present in the figure legend, table legend, main text, or Methods section.

n/a	Confirmed	
☐	☒	The exact sample size (*n*) for each experimental group/condition, given as a discrete number and unit of measurement
☐	☒	A statement on whether measurements were taken from distinct samples or whether the same sample was measured repeatedly
☐	☒	The statistical test(s) used AND whether they are one- or two-sided<br>*Only common tests should be described solely by name; describe more complex techniques in the Methods section.*
☐	☒	A description of all covariates tested
☐	☒	A description of any assumptions or corrections, such as tests of normality and adjustment for multiple comparisons
☐	☒	A full description of the statistical parameters including central tendency (e.g. means) or other basic estimates (e.g. regression coefficient) AND variation (e.g. standard deviation) or associated estimates of uncertainty (e.g. confidence intervals)
☐	☒	For null hypothesis testing, the test statistic (e.g. *F*, *t*, *r*) with confidence intervals, effect sizes, degrees of freedom and *P* value noted<br>*Give P values as exact values whenever suitable.*
☒	☐	For Bayesian analysis, information on the choice of priors and Markov chain Monte Carlo settings
☒	☐	For hierarchical and complex designs, identification of the appropriate level for tests and full reporting of outcomes
☒	☐	Estimates of effect sizes (e.g. Cohen's *d*, Pearson's *r*), indicating how they were calculated

Our web collection on statistics for biologists contains articles on many of the points above.

Software and code

Policy information about availability of computer code

Data collection	We did not develop software or codes for data collection.
Data analysis	We used R language. Relevant packages are cited in the main text.

For manuscripts utilizing custom algorithms or software that are central to the research but not yet described in published literature, software must be made available to editors and reviewers. We strongly encourage code deposition in a community repository (e.g. GitHub). See the Nature Portfolio guidelines for submitting code & software for further information.

Data

Policy information about availability of data

All manuscripts must include a data availability statement. This statement should provide the following information, where applicable:

- Accession codes, unique identifiers, or web links for publicly available datasets
- A description of any restrictions on data availability
- For clinical datasets or third party data, please ensure that the statement adheres to our policy

To reproduce figures, relevant data and R codes mentioned in the main text could be found in the following repository. Community weighted mean calculated from field raw data are included above. Field measured raw traits data will be open shared through Dryad and TRY trait database after peer-review. Figures, codes and data could be downloaded from https://github.com/Hzhang-ouce/Ghana_rainfall_trait_variation_optimality_github.

Research involving human participants, their data, or biological material

Policy information about studies with human participants or human data. See also policy information about sex, gender (identity/presentation), and sexual orientation and race, ethnicity and racism.

Reporting on sex and gender	Not involving human participants
Reporting on race, ethnicity, or other socially relevant groupings	Not involving human participants
Population characteristics	Not involving human participants
Recruitment	Not involving human participants
Ethics oversight	Not involving human participants

Note that full information on the approval of the study protocol must also be provided in the manuscript.

Field-specific reporting

Please select the one below that is the best fit for your research. If you are not sure, read the appropriate sections before making your selection.

☐ Life sciences ☐ Behavioural & social sciences ☒ Ecological, evolutionary & environmental sciences

For a reference copy of the document with all sections, see nature.com/documents/nr-reporting-summary-flat.pdf

Ecological, evolutionary & environmental sciences study design

All studies must disclose on these points even when the disclosure is negative.

Study description	We first summarized the expectations of recent 'universal' theories on how functional traits vary along wet-dry gradients in the tropic, which deduced 15 testable predictions. We focus on functional traits associated with plants' photosynthesis and water transport strategies. Plant functional traits were measured from intact forests/savanna, along a typical VPD gradient in West Africa that has strong variation in VPD and light but not temperatures.
Research sample	We selected three study areas in Ghana, West Africa. With in these study areas, we set up seven one-hectare forests monitoring plots. They are Ankasa (ANK, two moist rainforest plots), Bobiri (BOB, two semi-deciduous forest plots) and Kogyae (KOG, one dry forests plot and two mesic savanna plots).
Sampling strategy	We selected species that contributed to up to 80% of the basal area of each plot and sampled the three largest individuals for each species (see Appendix for exceptions). We then calculate a community weighted mean to represent overall status of the forests stand.
Data collection	Leaf traits field campaigns were conducted using a standardized protocol between October 2014 and September 2016 in all plots
Timing and spatial scale	Between October 2014 and September 2016, some traits are measured seasonally, see appendix for more details.
Data exclusions	Outliers were checked with the R package outliers::scores, interquartile range method (IQR) with threshold 1.5. Each extreme values are manually inspected. The values are kept when we were sure that they were devoid of error/typo.
Reproducibility	n/a (this study does not involve experiments, thus no repetition of experiments)
Randomization	For most of the traits, we have sampled a large number of leaves, please see figure one for the number of samples.
Blinding	n/a

Did the study involve field work? ☒ Yes ☐ No

Field work, collection and transport

Field conditions	Variables measuring the environment of the study sites are presented in Figure1 and Table S1.

Location	Ghana, West Africa, see coordinates in Table S1
Access & import/export	When exporting soil samples and leaves samples from Ghana, we applied for permits and adhered to relevant regulations.
Disturbance	Our plant functional traits measurements were made from clipped branches from wild trees.

Reporting for specific materials, systems and methods

We require information from authors about some types of materials, experimental systems and methods used in many studies. Here, indicate whether each material, system or method listed is relevant to your study. If you are not sure if a list item applies to your research, read the appropriate section before selecting a response.

Materials & experimental systems

n/a	Involved in the study
☒	☐ Antibodies
☒	☐ Eukaryotic cell lines
☒	☐ Palaeontology and archaeology
☒	☐ Animals and other organisms
☒	☐ Clinical data
☒	☐ Dual use research of concern
☐	☒ Plants

Methods

n/a	Involved in the study
☒	☐ ChIP-seq
☒	☐ Flow cytometry
☒	☐ MRI-based neuroimaging

Plants

Seed stocks	Our plant functional traits measurements were made from clipped branches from wild trees. We did not perform seeds germination.
Novel plant genotypes	N/A
Authentication	N/A
